# A general strategy for preparing pyrrolic-N_4_ type single-atom catalysts *via* pre-located isolated atoms

**DOI:** 10.1038/s41467-021-27143-5

**Published:** 2021-11-23

**Authors:** Junjie Li, Ya-fei Jiang, Qi Wang, Cong-Qiao Xu, Duojie Wu, Mohammad Norouzi Banis, Keegan R. Adair, Kieran Doyle-Davis, Debora Motta Meira, Y. Zou Finfrock, Weihan Li, Lei Zhang, Tsun-Kong Sham, Ruying Li, Ning Chen, Meng Gu, Jun Li, Xueliang Sun

**Affiliations:** 1 Department of Mechanical and Materials Engineering, University of Western Ontario, London, ON N6A 5B9 Canada; 2 Department of Chemistry, Southern University of Science and Technology, 518055 Shenzhen, China; 3 Department of Materials Science and Engineering, Southern University of Science and Technology, 518055 Shenzhen, China; 4 Science Division, Canadian Light Source Inc., 44 Innovation Boulevard, Saskatoon, SK S7N 2V3 Canada; 5 CLS @ APS, Advanced Photon Source, Argonne National Laboratory, Lemont, IL 60439 USA; 6 Department of Chemistry, University of Western Ontario, London, ON N6A 5B7 Canada; 7 Department of Chemistry and Key Laboratory of Organic Optoelectronics and Molecular Engineering of Ministry of Education, Tsinghua University, 100084 Beijing, China

**Keywords:** Renewable energy, Electrocatalysis, Catalyst synthesis

## Abstract

Single-atom catalysts (SACs) have been applied in many fields due to their superior catalytic performance. Because of the unique properties of the single-atom-site, using the single atoms as catalysts to synthesize SACs is promising. In this work, we have successfully achieved Co_1_ SAC using Pt_1_ atoms as catalysts. More importantly, this synthesis strategy can be extended to achieve Fe and Ni SACs as well. X-ray absorption spectroscopy (XAS) results demonstrate that the achieved Fe, Co, and Ni SACs are in a M_1_-pyrrolic N_4_ (M= Fe, Co, and Ni) structure. Density functional theory (DFT) studies show that the Co(Cp)_2_ dissociation is enhanced by Pt_1_ atoms, thus leading to the formation of Co_1_ atoms instead of nanoparticles. These SACs are also evaluated under hydrogen evolution reaction (HER) and oxygen evolution reaction (OER), and the nature of active sites under HER are unveiled by the *operando* XAS studies. These new findings extend the application fields of SACs to catalytic fabrication methodology, which is promising for the rational design of advanced SACs.

## Abstract (web summary)

Synthesizing single-atom catalysts through a general method presents a great challenge. Here the authors report that Fe, Co and Ni single-atom catalysts can be obtained using pre-located isolated Pt atoms as the catalyst and identify the role of Pt single atoms in the synthesis process.

## Introduction

Single-atom catalysts (SACs)^1^ have attracted considerable interest due to their superior catalytic activity and unique selectivity towards different chemical reactions, including CO oxidation^1,2^, hydrogenation^3^, dehydrogenation^4,5^, and electrochemical reactions^6–8^. Recently, the application of SACs has been widely extended to various research areas^9–14^ including energy storage systems like Li-S batteries^15^, enzyme catalysis^16^, photocatalysis^17^, and even cellular NO sensor^18^. Therefore, SACs are continuously showing great potentials for wide applications.

The most significant distinction between SACs and other types of catalysts is their unique single-atom-site, which both increases the atom utilization efficiency and tailors the interaction between the reactant and metal atoms through the adsorption and activation process. For example, Yan et al. reported the Pd_1_/graphene SACs showed a high butane selectivity in the hydrogenation of 1,3-butadiene, where the adsorption of 1,3-butadiene on Pd_1_ was mainly in a mono-π mode, which differs from that on Pd bulk^3^. Duchesne and co-workers showed that the Pt single atoms on Au nanoparticles (NPs) exhibited superior catalytic performance in formic acid oxidation. Such a high activity of Pt_4_Au_96_ is ascribed to the weakened CO adsorption on single and few Pt atoms than that on Pt bulk^19^. Li and co-workers demonstrated that the Pt_1_/Co_3_O_4_ exhibited a much weaker H_2_ adsorption energy than Pt bulk, thus leading to its high catalytic performance in the dehydrogenation of ammonia borane^5^.

However, because of their highly un-saturated coordination environment, heterogeneously supported single atoms possess a dramatically increased surface-free energy, which can lead to their aggregation into NPs, particularly at high metal loadings. Recently, some strategies have been developed to achieve relatively high-loading SACs. These methods include zeolite or mesoporous carbon to stabilize single atoms through a confinement strategy^20–23^, metal-organic frameworks (MOFs) to achieve SACs through high-temperature pyrolysis^24^, metal NPs to achieve SACs through high-temperature migration^25,26^, and a high-temperature shock wave treatment to achieve high loading SACs^27^. However, there are yet some restrictions on these newly developed methods such as general applicability. Therefore, developing a general approach for the synthesis of various types of SACs is highly desired.

Atomic layer deposition (ALD), a sequential surface reaction relying on a self-limiting process is used to synthesize non-noble single atoms such as Fe and Co^28,29^. Unfortunately, it results in a low metal loading and the formation of NPs, especially on carbon-based supports due to the high dissociation barrier and predominantly physical adsorption of metal precursors^28–31^. Therefore, a new reasonable route to produce SACs by ALD may be achieved by lowering the dissociation energy of the ALD precursor. For instance, this high dissociation energy might be strongly reduced on a metal surface^31–33^. Accordingly, the use of metal single atoms as the catalyst to facilitate the dissociation of ALD precursors to synthesize SACs would be very promising. To the best of our knowledge, this new SACs producing strategy that uses surface single atoms as the catalysts has never been reported. Moreover, the ALD method is feasible to obtain single atoms on various supports, which not only provides a new way to achieve SACs with dual atomic sites but also extends the application fields of SACs to the catalytic fabrication methodology.

In this work, we firstly verified the strategy by using supported Pt single atoms on N-doped carbon nanosheets (Pt_1_/NCNS) as the catalyst to synthesize Co SAC through ALD. X-ray absorption spectroscopy (XAS) results reveal singly dispersed Co_1_ atoms. Interestingly, the Pt_1_ atoms are still atomically dispersed after Co deposition, as confirmed by XAS and high-angle annular dark-field scanning transmission electron microscopy (HAADF-STEM). Furthermore, we also show that this synthesis strategy is general and can also be easily extended to achieve Fe and Ni SACs. X-ray absorption near edge structure (XANES) simulation and extended X-ray absorption fine structure (EXAFS) analysis results demonstrate that the achieved Co, Fe, and Ni SACs are in a M_1_-pyrrolic-N_4_ structure (M=Fe, Co, and Ni). Density functional theory (DFT) calculations show that Pt_1_ atoms promote the dissociation of Co(Cp)_2_ into CoCp and Cp fragments. The Co(Cp) product further deposited on the substrate through strong chemisorption, thus leading to a higher metal loading and formation of Co_1_ atoms. These SACs were evaluated under hydrogen evolution reaction (HER) and the nature of single-atom sites are unveiled by the *operando* XAS studies. Moreover, in the oxygen evolution reaction (OER), the Ni_1_ SAC showed much higher activity than Fe_1_ and Co_1_ SACs, which is in line with the DFT predictions.

## Results and discussion

### Direct fabrication of Pt and Co single atoms on NCNS using ALD

**Fig. 1:**
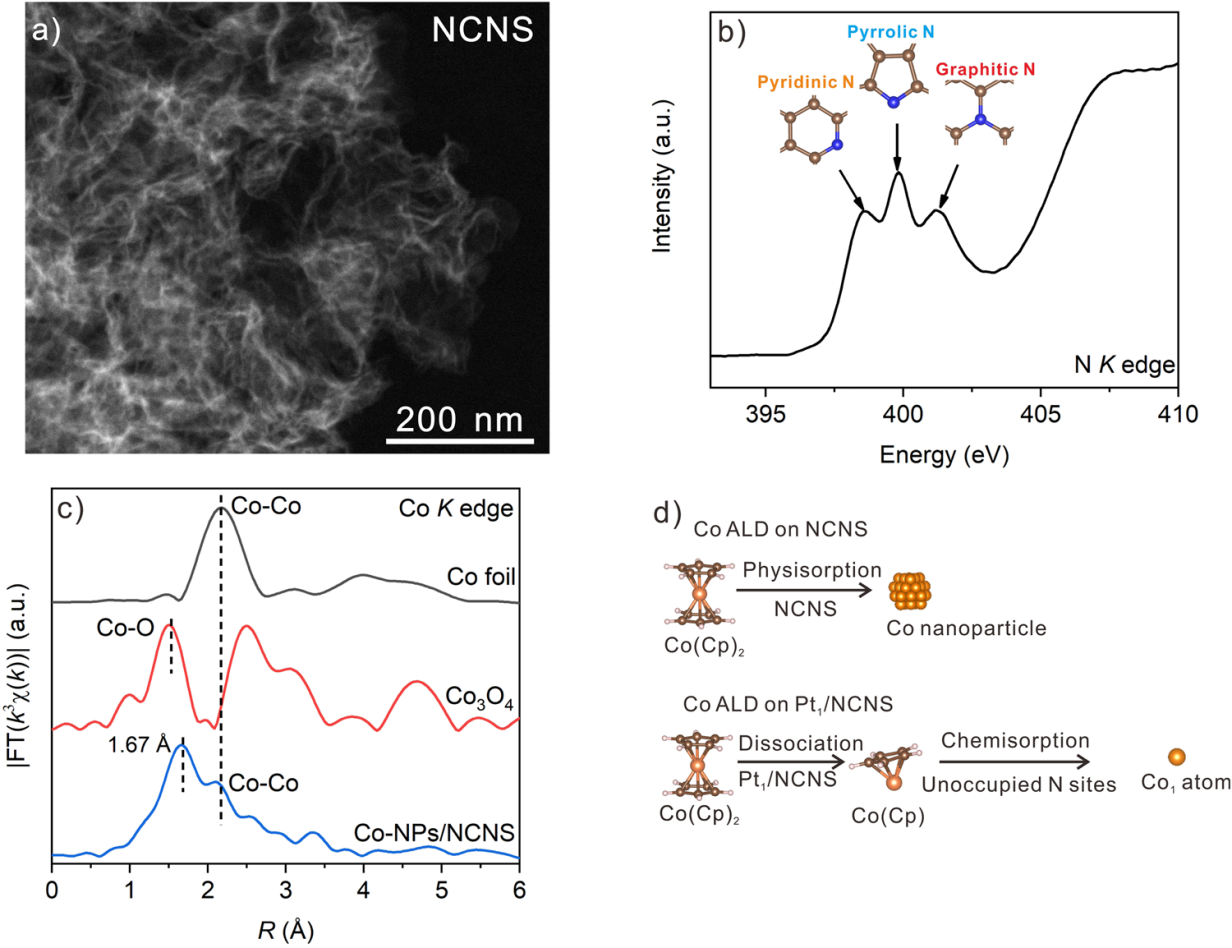
Characterizations for pristine NCNS, Co-NPs/NCNS, and proposed synthesis strategies for Co SAC. **a** HAADF-STEM image of NCNS. **b** XANES of NCNS at N *K* edge. **c** FT-EXAFS of Co-NPs/NCNS, as well as Co foil and Co_3_O_4_ reference at Co *K* edge, respectively. **d** Assumption of the Co ALD process on pristine NCNS and Pt_1_/NCNS. The white, brown, blue, and orange spheres represent H, C, N, and Co, respectively.

Graphene like NCNS (Fig. 1a and Supplementary Fig. 1) was firstly achieved using C_3_N_4_ as the template and glucose as the carbon source^34^. N *K*-edge XANES results indicate that there are various types of N species including pyridinic, pyrrolic, and graphitic N on NCNS (Fig. 1b)^35^. According to the literature, the N defects can coordinate with metal species and form a stable SAC^7^. Therefore, the NCNS substrate shows high potential for the synthesis of SACs. After performing one cycle of Pt ALD on NCNS, well-dispersed Pt single atoms were confirmed by HAADF-STEM (Supplementary Fig. 2) and XAS results (Supplementary Fig. 3)^36^. The loading of Pt is as high as 2.0 wt% based on the inductively coupled plasma optical emission spectrometer (ICP-OES) results.

Since the NCNS shows great potential to support Pt_1_ atoms, it has the possibility to act as a suitable substrate for other types of SACs. Using ALD, we performed one cycle of Co ALD on NCNS with Co(Cp)_2_ and O_2_ as the precursors. However, unlike the formation of Pt_1_ atoms, we found obvious Co NPs/clusters in HAADF-STEM (Supplementary Fig. 4a) and Co-Co coordination from the EXAFS results at the Co *K* edge (Fig. 1c), indicating the formation of Co NPs (Co-NPs/NCNS). Besides the formation of Co NPs, we also noticed a very low metal loading of Co (0.5 wt%). Such a low loading of Co indicates limited physisorption after the dose of Co(Cp)_2_ precursor, and the unreacted Co(Cp)_2_ would be purged away by N_2_ as illustrated in (Supplementary Fig. 5), leading to a low effective Co deposition^30^. Unfortunately, the weak adsorption effect of Co(Cp)_2_ on NCNS will form Co NPs due to the weak interaction between Co species and substrate when O_2_ is introduced. Therefore, the direct utilization of the Co ALD process to produce Co single atoms on NCNS is not practical.

### Synthesizing Co, Fe, and Ni SACs using pre-located isolated Pt_1_ atoms

**Fig. 2:**
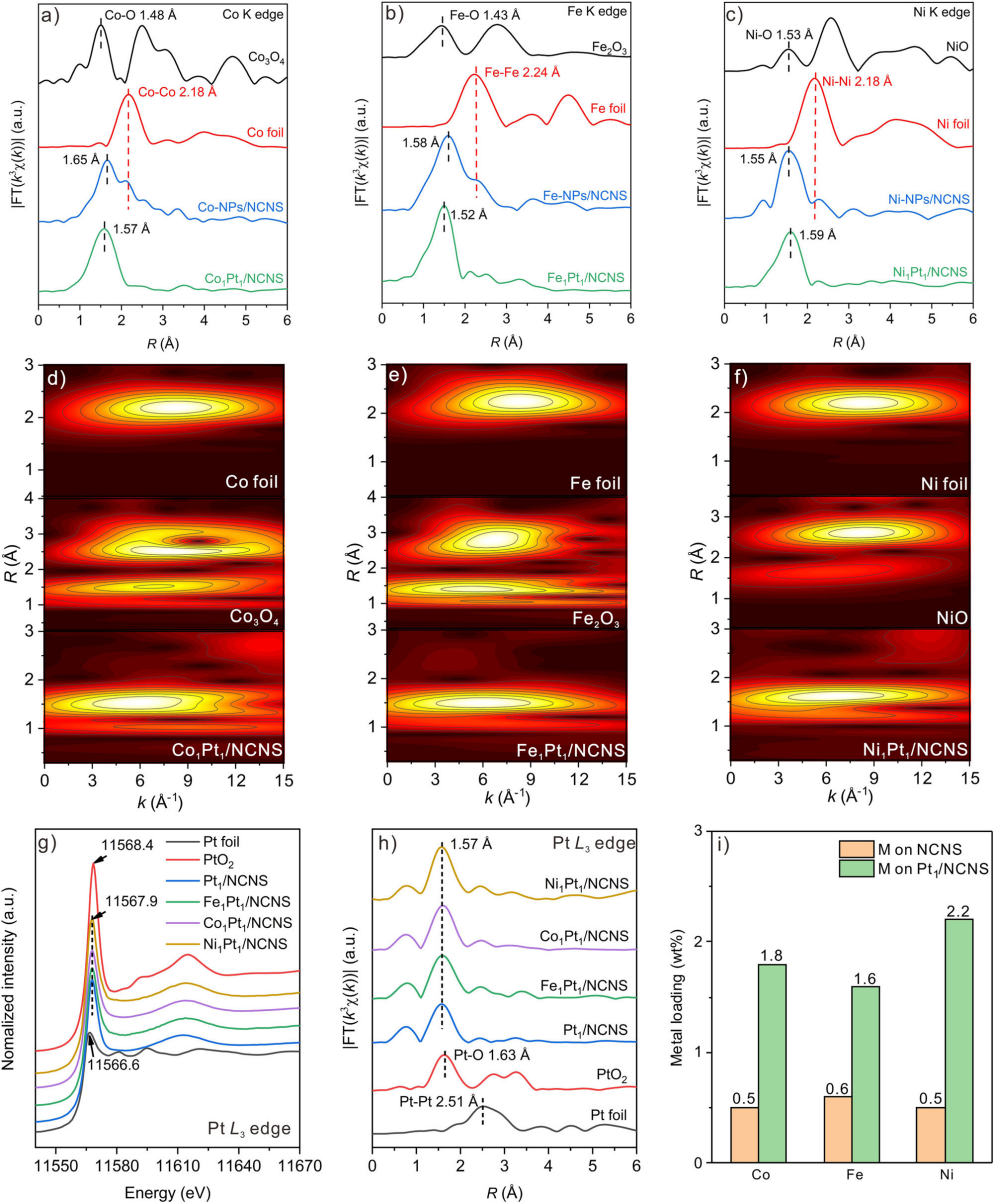
XAS characterizations for M_1_Pt_1_/NCNS catalysts. **a**–**c** Radial distribution curve from FT-EXAFS of Co-NPs/NCNS, Co_1_Pt_1_/NCNS, Fe-NPs/NCNS, Fe_1_Pt_1_/NCNS, Ni-NPs/NCNS, and Ni_1_Pt_1_/NCNS, respectively, as well as their corresponding foil and oxide reference at Co, Fe, and Ni *K* edges. **d**–**f** Wavelet-transformed spectra of Co_1_Pt_1_/NCNS, Fe_1_Pt_1_/NCNS, Ni_1_Pt_1_/NCNS, as well as their corresponding foil and oxide reference at Co, Fe, and Ni *K* edges. **g** and **h** XANES and FT-EXAFS of Pt_1_/NCNS, Co_1_Pt_1_/NCNS, Fe_1_Pt_1_/NCNS, and Ni_1_Pt_1_/NCNS, respectively, as well as Pt foil and PtO_2_ reference at Pt *L*_3_ edge. **i** ICP-OES results of M (Fe, Co, and Ni) metal loadings.

Inspired by previous research, the dissociation of Co(Cp)_2_ will be enhanced on the metal surface^37^. In our system, the Pt single atoms are easy to achieve, and the Pt-based catalysts are active in many chemical reactions. Therefore, the singly dispersed Pt_1_ atoms may have a similar ability and are likely capable to decrease the dissociation energy of Co(Cp)_2_, which may increase the Co loading and more importantly to achieve Co single atoms. With this idea in mind, we firstly conducted one cycle of Pt ALD on NCNS to achieve Pt_1_/NCNS and followed up with one cycle of Co ALD to verify our assumption (Fig. 1d). After performing one cycle of Co ALD on Pt_1_/NCNS substrate, the Co *K* edge EXAFS of this sample exhibits only one single peak at 1.57 Å (Fig. 2a), which can be attributed to the first shell of Co-C/N/O coordination. Meanwhile, the coordination of Co-Co is negligible, indicating that the Co species mostly exist as single atoms from the new synthesis strategy. In the following parts, this sample is denoted as Co_1_Pt_1_/NCNS.

In order to further extend the application of this synthesis strategy to other SACs such as Fe and Ni, we have chosen to use Fe and Ni ALD precursors with similar chemical structures as that of Co, i.e., ferrocene and nickelocene, respectively. Similarly, Fe and Ni single atoms also cannot be achieved by ALD on pristine NCNS as revealed by Fe-Fe and Ni-Ni coordination in the EXAFS (Fig. 2b, c) and TEM results (Supplementary Fig. 4). In contrast, the Fe-Fe and Ni-Ni coordination are significantly depressed when performing one cycle of Fe and Ni ALD on Pt_1_/NCNS (Fe_1_Pt_1_/NCNS and Ni_1_Pt_1_/NCNS) (Fig. 2b, c). Wavelet-transform EXAFS results further verified the atomically dispersed Co, Fe, and Ni single atoms, which differs from that of their foil and oxide (Fig. 2d, f).

More interestingly, the Pt *L*_*3*_ edge XANES (Fig. 2g) and X-ray Photoelectron Spectroscopy (XPS) (Supplementary Fig. 6) results show inconspicuous differences, indicating the electronic properties of Pt on these M_1_Pt_1_/NCNS (M=Fe, Co, and Ni) samples are close to that on Pt_1_/NCNS. Pt *L*_*3*_ edge EXAFS results further confirm the stable local structure of the Pt_1_ atom (Fig. 2h). Typical EXAFS fitting on Co_1_Pt_1_/NCNS at Pt *L*_*3*_ edge also demonstrates the unchanged atomic nature of Pt single atoms (Supplementary Fig. 7 and Supplementary Table 1). These results indicate that the Pt_1_ atoms are still atomically dispersed after the deposition of Co, Fe, and Ni. This observation provides strong evidence that the Pt_1_ atoms only act as the catalyst, while they change the deposition process of Co, Fe, and Ni, but does not lose the atomic dispersion or form a Pt-Co/Fe/Ni cluster, because of the lack of metal-metal coordination in Pt *L*_*3*_, Fe, Co, and Ni *K* edges EXAFS results. Subsequently, we examined the Co loading through ICP-OES after performing one cycle of Co ALD on pristine NCNS and Pt_1_/NCNS (Fig. 2i). The ICP-OES results show that the Co loading of Co_1_Pt_1_/NCNS (1.8 wt%) is more than three times higher than that of Co-NPs/NCNS (0.5 wt%). Similar results are also observed on Fe and Ni, showing low Fe and Ni loadings on Fe-NPs/NCNS and Ni-NPs/NCNS (0.6 and 0.5 wt%) but much higher on Fe_1_Pt_1_/NCNS and Ni_1_Pt_1_/NCNS (1.6 and 2.2 wt%), respectively. These results unambiguously demonstrate that the Pt_1_ atoms change the adsorption model of M precursors to predominantly chemisorption, which promotes the degree of effective deposition. However, when the M ALD was performed on pristine NCNS, physisorption is dominant, thus leading to a low metal loading because of less effective deposition.

### Morphology of M_1_Pt_1_/NCNS catalysts

**Fig. 3:**
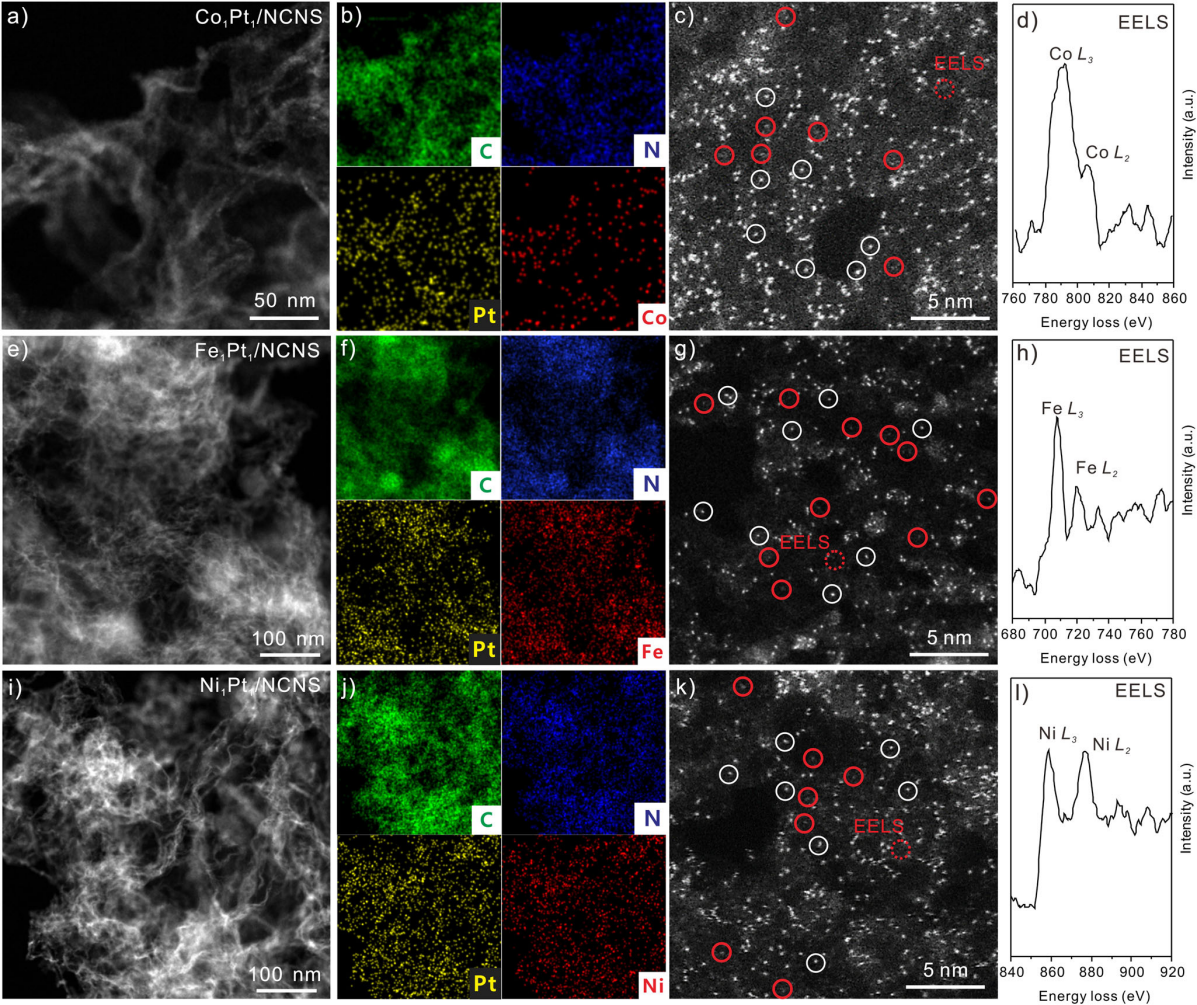
Morphology of M_1_Pt_1_/NCNS catalysts. HAADF-STEM images of Co_1_Pt_1_/NCNS (**a**), Fe_1_Pt_1_/NCNS (**e**), and Ni_1_Pt_1_/NCNS (**i**) at low resolution and corresponding EDX mapping (**b**, **f**, and **j**). HAADF-STEM images on Co_1_Pt_1_/NCNS (**c**), Fe_1_Pt_1_/NCNS (**g**), and Ni_1_Pt_1_/NCNS (**k**) samples. The white circles highlight the Pt single atoms. The red circles highlight the Co, Fe, and Ni single atoms, respectively. **d**, **h**, and **l** Corresponding EELS spectra at the location of red dash circle in **c**, **g**, and **k**.

The morphology of M_1_Pt_1_/NCNS catalysts is characterized by HAADF-STEM. As shown in Fig. 3a, e, i, no observable Pt or M NPs can be found at low magnification. Energy dispersive X-ray (EDX) mapping results show the Pt and M elements are well-dispersed on the NCNS substrate (Fig. 3b, f, j and Supplementary Figs. 8 to 10), indicating their uniform dispersion and high density. At high magnification, high-density bright Pt single atoms (white circles) can be found on Co_1_Pt_1_/NCNS (Fig. 3c and Supplementary Fig. 8f), Fe_1_Pt_1_/NCNS (Fig. 3g and Supplementary Fig. 9f), and Ni_1_Pt_1_/NCNS (Fig. 3k and Supplementary Fig. 10f). All the visible Pt atoms are singly dispersed without any obvious clusters or NPs. Meanwhile, some isolated atoms with less brightness (red circles) were also found to reside nearby the bright Pt_1_ atoms, which can be assigned to Fe, Co, and Ni single atoms due to their lower Z-contrast. Furthermore, when the electron energy loss spectroscopy (EELS) was measured at the atoms with less brightness on Co_1_Pt_1_/NCNS, the signals of Co *L*_*2*_ and *L*_*3*_ could also be detected (Fig. 3d), confirming the existence of Co_1_ atoms. Similar results can be also found on Fe_1_Pt_1_/NCNS (Fig. 3h) and Ni_1_Pt_1_/NCNS (Fig. 3i). Based on the HAADF-STEM results, the Fe, Co, and Ni-Pt pairs are absent. Taken together, the EXAFS and HAADF-STEM results demonstrate the single-atom nature of both Pt and M atoms on NCNS.

### Structure identification of M SACs

**Fig. 4:**
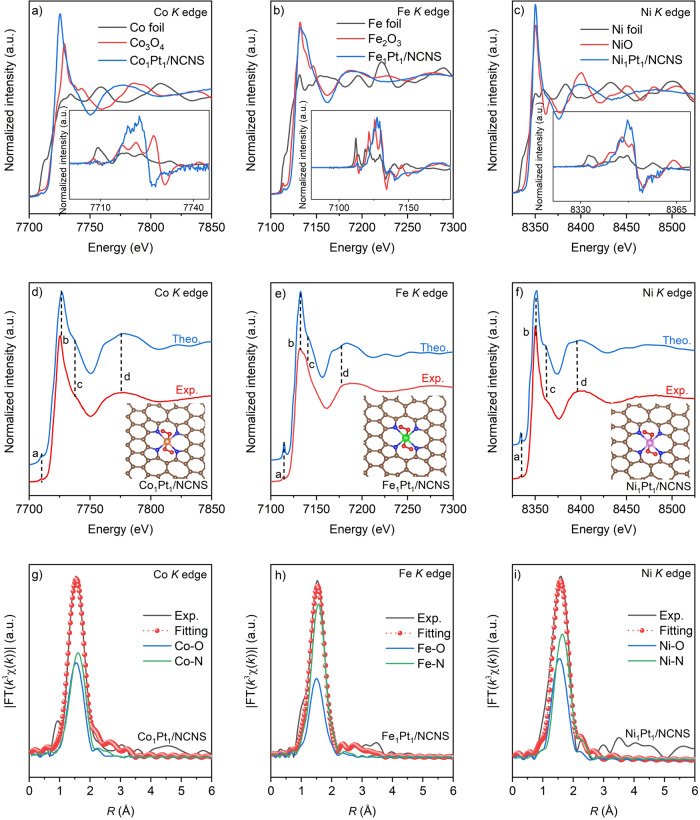
Identification of the local structure of M_1_Pt_1_/NCNS catalysts. **a**–**c** The experimental *K*-edge XANES spectra and first derivative curves (insets) of M_1_Pt_1_/NCNS and references samples. **d**–**f** Comparison between the experimental *K*-edge XANES spectra of M_1_Pt_1_/NCNS and the simulated spectra. Some of the main features reproduced are highlighted at points “a” to “d”. Fitting results of the *k*^3^-weighted FT spectrum of M_1_Pt_1_/NCNS at Co (**g**), Fe (**h**), and Ni (**i**) K edges. The white, brown, blue, red, orange, green, and pink spheres represent H, C, N, O, Co, Fe, and Ni, respectively.

Using this synthesis strategy, we have successfully achieved Co, Fe, and Ni SACs. Unveiling the local structure of these SACs is critical to understand the synthesis mechanism and their catalytic applications. XANES spectroscopy is very sensitive to the local geometric structure of the photon-absorbing atom, which provides a better option to understand the local atomic structure. As shown in Fig. 4a to c, the Co_1_Pt_1_/NCNS, Fe_1_Pt_1_/NCNS, and Ni_1_Pt_1_/NCNS at Co, Fe, and Ni *K* edge XANES spectra, respectively, show significant differences from their corresponding foil and oxide references. We also notice that the XANES spectra of Co_1_Pt_1_/NCNS, Fe_1_Pt_1_/NCNS, and Ni_1_Pt_1_/NCNS are nearly identical, indicating a similar local coordination environment of M_1_Pt_1_/NCNS catalysts. This can be delineated since the k-dependent absorber phase shift of Fe, Co, and Ni, adjacent in the periodic table, is very similar. Taking the first derivative XANES of M_1_Pt_1_/NCNS, the energy position of the point of first inflection suggests that the oxidation states of Fe, Co, and Ni are close to +3, +2, and +2, respectively. These observations are perfectly in line with the XPS results (Supplementary Fig. 11).

Focusing on the Co_1_Pt_1_/NCNS catalyst, we first compared the modeling XANES result based on a recently reported Co-C_4_-O_2_ type Co_1_ atom on graphene using ALD (Supplementary Fig. 12a)^38^. Significant differences can be observed between the theoretical simulated XANES and experimental results, indicating the necessity to consider the critical role of N atoms. We then further compared the reported pyridinic-N_4_-based Co_1_-N_4_ structure, which still shows the obvious difference with our experimental XANES results (Supplementary Fig. 12b), suggesting the existence of defects in this model as well. Then we progressively add one and two end-on oxygen moieties along the axial direction of the pyridinic Co_1_-N_4_ structure (Supplementary Fig. 12c, d). Although parts of the experimentally resolved features can be reproduced and achieve a satisfactory agreement with the Co_1_Pt_1_/NCNS Co *K* edge XANES results, the short Co-N bonding distance (less than 1.90 Å) disagrees with our EXAFS results (Fig. 2a). Considering that there is a large proportion of pyrrolic-N in the NCNS support, the possibility of a pyrrolic-N_4_ type Co_1_-N_4_ structure cannot be ruled out. As shown in Supplementary Fig. 12e, the simulated XANES result of pyrrolic-N_4_-based Co_1_-N_4_ showed much better conformity than the pyridinic-N_4_ structure. We then further added one molecule of dioxygen with end on coordination on Co_1_ atom, the features from “b” to “d” are well reproduced but the pre-edge feature “a” is exaggerated (Supplementary Fig. 12f). However, when we added another molecule of dioxygen on the Co_1_ atom at the opposite side, all the features from “a” to “d” can be correctly reproduced (Fig. 4d). Similarly, the simulated 2O_2_-Fe_1_-pyrrolic N_4_ and 2O_2_-Ni_1_-pyrrolic N_4_ XANES spectra (Fig. 4e, f) show the most satisfactory results comparing with other DFT optimized structures (Supplementary Figs. 13 and 14). It is important to note, this is the first time that general M_1_-pyrrolic N_4_ type SACs can be achieved using ALD, as this type of SACs is usually achieved through high-temperature pyrolysis and ball-milling methods^39,40^. Bedsides the above XANES simulation, we also carried out the EXAFS fitting and the results consistently demonstrate the 2O_2_-M_1_-pyrrolic N_4_ moiety (Fig. 4g to i, Supplementary Figs. 15 to 18, and Table 1). It is worth noting that the EXAFS results perfectly match the DFT results (Supplementary Table 2). In summary, a close local structure of Co_1_Pt_1_/NCNS, Fe_1_Pt_1_/NCNS and Ni_1_Pt_1_/NCNS from simulated XANES and EXAFS fitting results strongly suggest the same deposition mechanism for Co, Fe, and Ni ALD on Pt_1_/NCNS, which indicates that this synthesis strategy can be a universal approach to achieve M_1_-pyrrolic N_4_ type SACs using Pt_1_ atoms.

**Table 1:** Structural parameters of the M_1_Pt_1_/NCNS (M=Fe, Co, and Ni), foil, and oxide references extracted from quantitative EXAFS curve-fittings at Co, Fe, and Ni *K* edges.

Sample	Path	CNs	*R* (Å)	σ^2^ (10^−3^Å^2^)	Δ*E*_0_ (eV)
Co foil	Co-Co	12.0	2.49	6.1	6.9
Co_3_O_4_	Co-O	4.0	1.91	1.7	1.0
Co_1_Pt_1_/NCNS	Co-O	2.0	1.98	3.3	2.2
	Co-N	4.0	2.08	6.4	
Fe foil	Fe-Fe	8.0	2.46	5.3	5.3
	Fe-Fe	6.0	2.85		
Fe_2_O_3_	Fe-O	6.0	1.93	10.0	−6.0
Fe_1_Pt_1_/NCNS	Fe-O	2.2	1.95	2.0	−4.2
	Fe-N	4.2	2.02	2.9	
Ni foil	Ni-Ni	12.0	2.48	6.1	7.4
NiO	Ni-O	6.0	2.05	4.2	−3.7
Ni_1_Pt_1_/NCNS	Ni-O	2.2	1.99	1.5	2.1
	Ni-N	4.2	2.11	2.5	

*CNs* coordination numbers, *R* bonding distance, *σ*^*2*^ Debye-Waller factor, Δ*E*_*0*_ inner potential shift. Errors in the fitting parameters are CN ± 20%, R ± 0.02, *σ*^2^ ± 20%, and Δ*E*_0_ ± 3.0.

### Theoretical understanding of deposition process

**Fig. 5:**
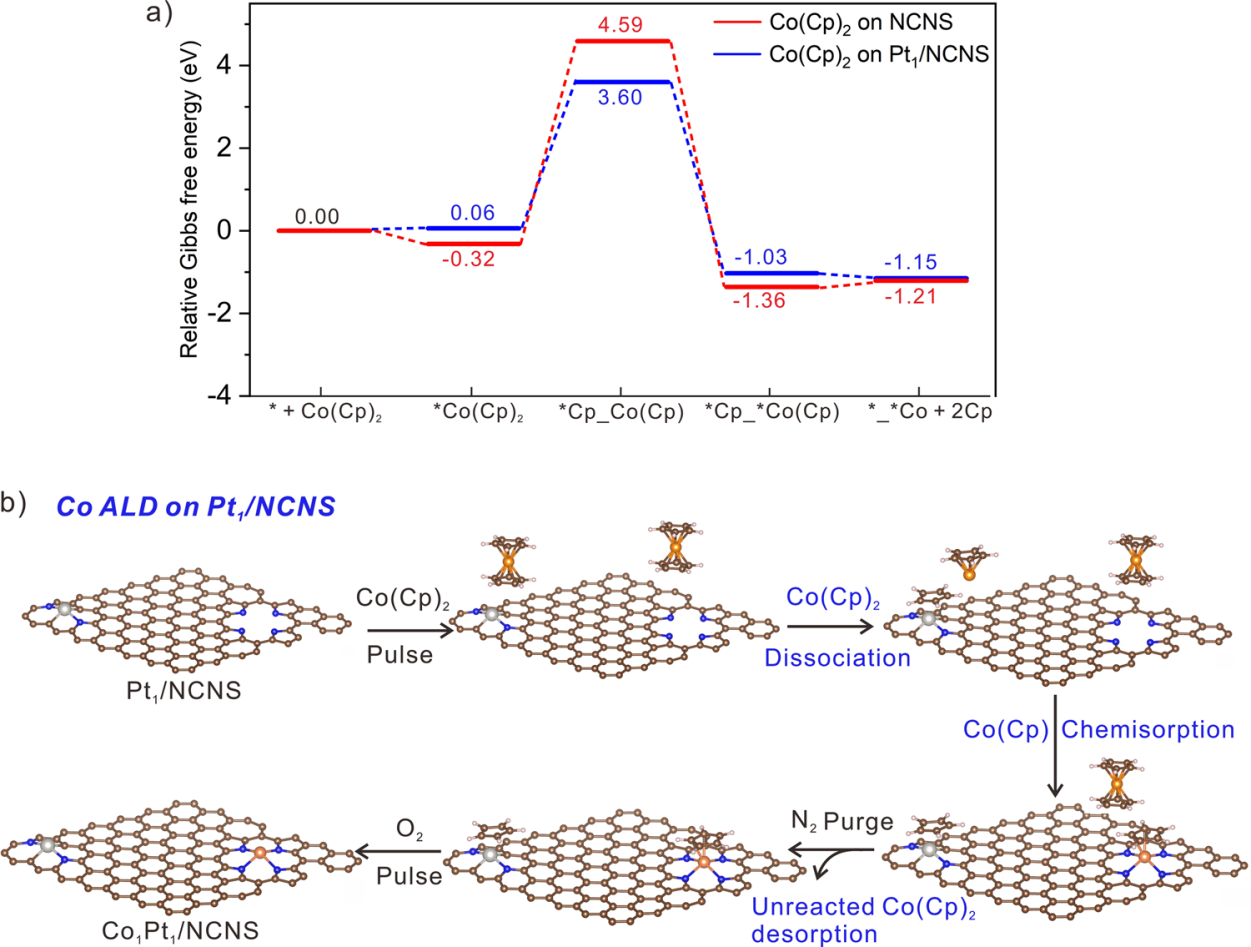
Illustration of theoretical understanding of the Co ALD process. **a** Theoretical reaction coordinate for Co(Cp)_2_ deposition on pristine NCNS and Pt_1_/NCNS. The Gibbs free energies were calculated at T = 523 K and P = 1 atm. **b** Illustration of Co ALD process on Pt_1_/NCNS. The white, brown, blue, orange, and silver spheres represent H, C, N, Co, and Pt, respectively.

Having resolved the structures of M_1_Pt_1_/NCNS and Pt_1_/NCNS, we carried out DFT calculations to elucidate the deposition mechanism. Considering the similar structure of Co, Fe, and Ni ALD precursors and local structures of Co_1_Pt_1_/NCNS, Fe_1_Pt_1_/NCNS, and Ni_1_Pt_1_/NCNS catalysts, typical Co ALD process is chosen for the following theoretical investigation. As revealed by the calculated Gibbs free energies shown in Fig. 5a, the Co(Cp)_2_ has moderate adsorption (Δ*G*_ad_ = −0.32 eV) but very high dissociation energy (Δ*G*_de_ = 4.91 eV) on the pristine NCNS. Therefore, the physisorption of Co(Cp)_2_ should be dominant on pristine NCNS. This physically and weakly adsorbed Co(Cp)_2_ will lead to a low metal loading from inefficient deposition (Fig. 2i and Supplementary Fig. 5). Owing to the weak physisorption, Co species will easily aggregate because of the lack of strong chemical bonding with the substrate. Therefore, Co NPs were observed in the direct Co ALD process on NCNS (Fig. 1c). However, when Pt_1_ atoms are involved in the Co ALD process, the deposition model differs considerably. Although the adsorption of Co(Cp)_2_ on Pt_1_ atom is slightly weaker (Δ*G*_ad_ = +0.06 eV), the dissociation energy of Co(Cp)_2_ is reduced to Δ*G*_de_ = 3.54 eV. The lower dissociation energy can be attributed to the electron transfer from the Cp ligand to the Pt_1_/NCNS, thus weakening the interactions between Co and Cp, which facilitates the dissociation process (Supplementary Fig. 19 and Supplementary Table 3). Considering it is a gas phase reaction, the Co(Cp) likely diffuses and deposits on the unoccupied pyrrolic N_4_ sites through chemisorption. Such a strong chemical bonding of Co with pyrrolic N_4_ sites will form a stable Co_1_ structure after the O_2_ pulse to remove the Cp ligand. It is worth noting that the introduction of O_2_ may also remove the attached Cp ligand on the Pt_1_ atom and recover the structure of Pt_1_/NCNS as shown in the Pt *L*_*3*_ XANES and EXAFS results (Fig. 2g, h). As a result, when the Co ALD is performed on Pt_1_/NCNS, Co single atoms will form and Pt_1_ atoms will remain atomic dispersion (Fig. 5b). The principle revealed by the theoretical calculation results indicates that this synthesis strategy could be universal for the rational design of SACs (Supplementary Fig. 20).

### Theoretical simulation and electrocatalytic performance of M_1_Pt_1_/NCNS

**Fig. 6:**
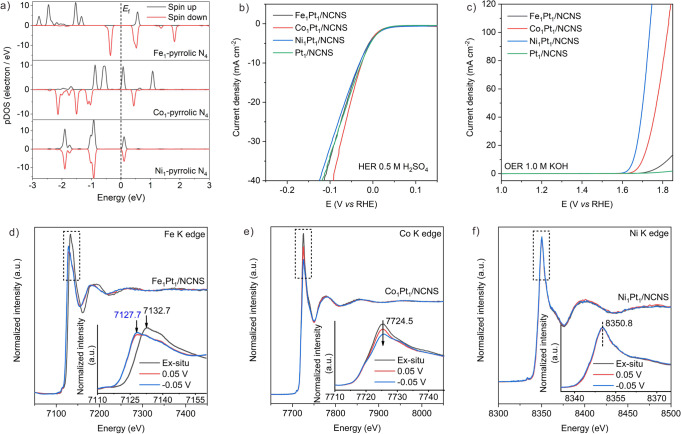
Theoretical and experimental electrocatalytic analysis on M_1_Pt_1_/NCNS (M=Co, Fe, and Ni). **a** Projected density of state (pDOS) on the M_1_ 3*d* orbitals with the Fermi level set at zero. HER (**b**) and OER (**c**) LSV curves of M_1_Pt_1_/NCNS and Pt_1_/NCNS with IR correction. **d**–**f** *Operando* XANES study on M_1_Pt_1_/NCNS catalysts in HER working conditions under different potentials at Fe, Co, and Ni K edges, respectively. Inserts: the enlarged area of XANES region in the dash square.

The capability of application for the achieved M_1_Pt_1_/NCNS catalysts was further evaluated in electrochemical reactions. Prior to the experiment, we first studied the projected density of state (pDOS) of various M_1_-pyrrolic N_4_ sites. As shown in Fig. 6a, the Co_1_-pyrrolic N_4_ and Fe_1_-pyrrolic N_4_ show higher DOS near the Fermi level than that of Ni_1_-pyrrolic N_4_. As is known, the high electron densities near the Fermi level could facilitate the adsorption of the proton (H^+^)^41–43^. Further calculated hydrogen adsorption free energy on Co_1_-pyrrolic N_4_ catalyst is closer to zero than that on Fe_1_-pyrrolic N_4_, indicating its relatively higher catalytic performance in HER (Supplementary Fig. 21). The Ni_1_-pyrrolic N_4_ shows the weakest H adsorption ability, indicating its unsatisfactory HER activity. This theoretical prediction is confirmed by the experiment, in which the Co_1_Pt_1_/NCNS showed higher activity than Fe_1_Pt_1_/NCNS and Ni_1_Pt_1_/NCNS (Fig. 6b). The higher catalytic activity of Co_1_Pt_1_/NCNS than the Pt_1_/NCNS could be attributed to the contributions of Co_1_ atoms in HER (Supplementary Figs. 22 and 23)^44–46^. The Co_1_Pt_1_/NCNS also exhibits good stability without showing any obvious activity decrease after 8,000 cycles durability test (Supplementary Fig. 24). Although the Ni_1_-pyrrolic N_4_ site exhibits low catalytic activity in HER, the high *d*-band center near the Fermi level on the Ni_1_ atom (Fig. 6a and Supplementary Table 4) shows the promising application in OER^47^. In the experiment, the Ni_1_Pt_1_/NCNS shows the best catalytic performance among various SACs in OER (Fig. 6c), which confirms the theoretical predication (Supplementary Figs. 25 and 26). The NCNS shows no catalytic activity in OER, indicating the carbon corrosion is not obvious (Supplementary Fig. 27).

The non-noble SACs are reported to show promising HER performance but the nature of active sites under HER has not been systematically investigated yet. Therefore, in order to better understand the nature of single-atom sites under reaction, *operando* XAS experiments were conducted on M_1_Pt_1_/NCNS under HER at Fe, Co, and Ni *K* edges, respectively (Supplementary Fig. 28). As shown in Fig. 6d, a distinct energy chemical shift in the XANES white-line (WL) region can be observed on Fe_1_Pt_1_/NCNS at Fe *K* edge from 7132.7 to 7127.7 eV when the overpotential is applied on 0.05 V. This *operando* XANES results indicate the reduced oxidation state of Fe from +3 to +2. This Fe^3+^(d^5^)/Fe^2+^(d^6^) redox transition is probably from the desorption of covered O species or impressed voltage, which would lead to a distorted Fe^2+^-N_4_ structure^40,48^. As shown in Fig. 6e, an obvious WL intensity decrease can be found on the Co_1_Pt_1_/NCNS at Co *K* edge with the increasing overpotentials. Unlike the results of Fe (Supplementary Fig. 29a), the E_0_ on the Co *K* edge keeps constant (Supplementary Fig. 29b). This decreased WL intensity indicates the filling of *p* electrons on Co, which could play an important role in the HER. The constant E_0_ is likely from the charge redistribution from the hybridization among atomic orbitals in Co, which results in the negligible net effect on E_0_. In sharp contrast with the *operando* XANES results on Fe_1_ and Co_1_ atoms, neither the E_0_ nor the WL intensity on Ni *K* edge of Ni_1_ atoms showed obvious changes (Fig. 6f and Supplementary Fig. 29c), indicating the Ni_1_-pyrrolic N_4_ sites may be intact under HER. We also measured the operando XANES on the Fe, Co, and Ni -NPs/NCNS under HER for comparison, where no changes can be found on all the spectra (Supplementary Fig. 30). These apparently insensitive responses under electrochemical reactions might account for their poor activity in HER (Supplementary Fig. 31).

For the first time, the synthesis of M_1_-pyrrolic N_4_ type SACs has been successfully achieved using heterogeneously supported Pt_1_ atoms as catalysts. This general M_1_-pyrrolic N_4_ framework is confirmed by XANES simulation and EXAFS analysis. DFT calculations show that the Pt_1_ atoms act as the catalyst to modulate the adsorption behavior and promote the dissociation of Co(Cp)_2_ in Co ALD, which leads to the chemical binding of CoCp on the pyrrolic N_4_ site and formation of Co single atoms. More importantly, this synthesis strategy can be extended to achieve Fe and Ni SACs. These SACs are evaluated under HER and the nature of single-atom sites are further unveiled by the operando XAS studies. In OER, the Ni_1_Pt_1_/NCNS catalyst show much better catalytic activity than the Fe and Co SACs counterparts, which is in excellent agreement with DFT prediction. These new findings extend the application fields of SACs to catalytic fabrication methodology, which is promising for the rational design of advanced SACs. The prominent covalent metal-support interaction bonding of M_1_ atom (M=Fe, Co, Ni) with the local coordinated N-atoms provides a unique opportunity to further tune the catalytic properties through substrate design^49,50^.

## Methods

### Synthesis of g-C_3_N_4_

Twelve grams of urea was put into an alumina crucible with a cover and then heated to 580 °C at a rate of 3 °C/min in a muffle furnace and maintained at this temperature for 4 h. After cooling down to room temperature, ~600 mg of bulk g-C_3_N_4_ was obtained.

### Synthesis of N-doped carbon nanosheet

The synthesis method for the N-doped carbon nanosheets is based on a previous report^34^. Typically, 500 mg g-C_3_N_4_ was mixed with 2.16 g glucose and dispersed in 40 mL of deionized water under sonication for 5 h. After that, the suspension was transferred to a Teflon autoclave and heated at 140 °C for 11 h in an oven. The product was collected by centrifugation and washed with water and ethanol several times, then dried under vacuum at 60 °C overnight. After that, the dried powder was heated up to 900 °C in Ar at a rate of 5 °C/min and maintained at this temperature for 1 h to achieve the N-doped carbon nanosheet (NCNS).

### Synthesis of Pt_1_/NCNS

Pt was deposited on the as-prepared NCNS by ALD (Savannah 100, Cambridge Nanotechnology Inc., USA) using trimethyl(methylcyclopentadienyl)-platinum (IV) (MeCpPtMe_3_) and O_2_ as precursors. High-purity N_2_ (99.9995%) was used as both a purging gas and carrier gas. The deposition temperature was kept at 150 °C, while the container for MeCpPtMe_3_ was heated to 65 °C to provide a steady flux of Pt to the reactor. The manifold was kept at 115 °C to avoid any condensation^5^. The timing sequence of one cycle Pt ALD was 15s and 30s for MeCpPtMe_3_ exposure and N_2_ purge respectively. Subsequently, the ALD chamber was heated up to 250 °C, with 30s O_2_ exposure and 30s N_2_ purge to remove the ligand.

### Synthesis of M_1_Pt_1_/NCNS (M=Fe, Co, and Ni)

The M ALD is conducted on the as-prepared Pt_1_/NCNS. The precursor used for Co, Fe and Ni are Ferrocene (Fe(Cp)_2_), Cobaltocene (Co(Cp)_2_) and Nickelocene (Ni(Cp)_2_). High-purity N_2_ (99.9995%) was used as both a purging gas and carrier gas. The deposition temperature was kept at 250 °C, while the container for M(Cp)_2_ was heated to 90 °C to provide a steady flux of precursor to the reactor. The manifold was kept at 140 °C to avoid any condensation. The timing sequence of one cycle M ALD was 15s and 30s for M(Cp)_2_ exposure and N_2_ purge respectively. Subsequently, the ALD chamber was heated up to 300 °C, with 30s O_2_ exposure and 30s N_2_ purge to remove the ligand.

### Instrumentation

TEM samples were prepared by drop-casting an ultrasonicated solution of dilute high-performance liquid chromatography grade methanol solution with the sample of interest onto a lacey carbon grid. The TEM characterization was carried out on a FEI Titan Cubed 80–300 kV microscope equipped with spherical aberration correctors (for probe and image forming lenses) at 200 kV. Atomic-resolution high-angle annular dark-field scanning transmission electron microscopy (HAADF-STEM) images were taken using a double spherical aberration-corrected FEI Themis microscope operated at 300 kV. The corresponding inner and outer collection semi-angles for HAADF imaging were 48–200 mrad. Dual EELS spectrum imaging was performed at a collection semi-angle of 28.7 mrad with a dispersion of 0.5 eV/channel. The metal loadings were analyzed using an inductively coupled plasma optical emission spectrometer (ICP-OES) with samples dissolved in hot fresh aqua regia overnight and filtered.

### Electrochemical measurements

The electrochemical measurements were performed using a glassy carbon rotating-disk electrode (Pine Instruments) as the working electrode with carbon paper and a standard hydrogen electrode as the counter and reference electrodes, respectively. Ink for the electrochemical measurement was prepared by adding 2 mg of the catalyst into 1 mL ethanol, and Nafion (5% solution, Sigma-Aldrich, 20 μL), followed by sonication for 10 min. A working electrode was prepared by loading the ink (10 μL) on the glassy carbon electrode. The HER test was carried out in 0.5 M H_2_SO_4_ with a scan rate of 0.01 V s^−1^. The durability test was carried out in 0.5 M H_2_SO4 between −0.1 and 0.4  V at a scan rate of 0.1 V s^−1^. The OER LSV polarization curves were measured in a O_2_-saturated electrolyte at a sweep rate of 5 mV s^−1^. The durability test was carried out on a constant current density of 10 mA/cm^2^. The measured potential against the reference electrode was converted to a reversible hydrogen electrode (RHE).

### XAS measurements

XAS measurements were conducted on the 061D superconducting wiggler at the hard X-ray microanalysis (HXMA) beamline at the Canadian Light Source (CLS) and beamline 20-ID-C at the CLS@APS of the Advanced Photon Source (APS), Argonne National Laboratory (ANL).

At HXMA beamline, each spectrum was collected using fluorescence yield mode with a Canberra 32 element Ge detector. The beamline initial energy calibration for the different edges was made by using the corresponding metallic foils from Exafs Materials, and the same metallic foil was further set between two FMB Oxford ion chamber detectors downstream to the sample, making in-step energy calibration available for each individual XAFS scan.

At 20-ID-C beamline, the XAS measurements were conducted at A Si (111) fixed-exit, double-crystal monochromator was used. Harmonic rejection was facilitated by detuning the beam intensity 15% at ∼1000 eV above the edge of interest. The measurements were performed in fluorescence mode using a four-element Vortex Si Drift detector. Details on the beamline optics and instruments can be found elsewhere^51^.

*Operando* XAFS measurements were performed with catalyst-coated carbon paper using a custom-built cell. The carbon paper was pretreated with concentrated nitric acid at 80 °C overnight to ensure thorough electrolyte wetting. The catalyst ink was prepared with the same method as for the electrochemical measurements. The catalyst ink was dropped onto the carbon paper, and the backside of the carbon paper was taped with the Kapton film as the working electrode to ensure the entirety of the electrocatalyst had access to the H_2_SO_4_ electrolyte. Carbon paper and Ag/AgCl were used as the counter and reference electrodes. To collect the XAFS spectra during the HER process, the cathodic voltages were held at constant potentials during the operando experiment from 0.05 to −0.05 V (*vs* RHE), respectively.

### XANES modeling

To further understand the nature of experimental resolved XANES features and address them to the structural and chemical nature of M (Fe, Co, and Ni) site occupancy and their corresponding local structural environment, DFT guided XANES theoretical modeling was carried out by using code FDMNES, following standard procedure^52^. For instance, a Co centered cluster was developed based on the structure model predicated by DFT optimized structures. The radius of the cluster is around 6 Å, corresponding roughly to the detection limit of the XAFS data.

### EXAFS data analysis

The data reduction was using codes Athena^53^. The followed EXAFS R space curve fitting was performed using WINXAS version 2.3, following the procedure reported reference^54^. In brief, the first inflection point of the XANES edge was defined as the experimental E_0_, the post-absorption edge background was estimated and removed by cubic spline fits. Based on the local structural environment predicted by DFT theoretical modeling, the scattering amplitudes and phases were calculated using FEFF7.02 and further used for R space curve fitting^55^. The k rages for FT-EXAFS for Pt *L*_*3*_, Fe, Co, and Ni *K* edges are 3.15–11.57, 2.50–11.16, 2.37–10.90, and 2.33 to 10.48 Å^−1^, respectively.

### Theoretical and computational methods

First-principles DFT calculations were performed by using the spin-polarized Kohn–Sham formalism with the generalized gradient approximation (GGA) of Perdew-Burke-Ernzerhof (PBE)^56^, as implemented in the Vienna ab initio Simulation Package (VASP 5.4.4)^57^. The valence electronic states of all atoms were expanded in plane-wave basis sets with a cutoff energy of 400 eV, and gamma points were used for Brillouin zone integration. All atoms were allowed to relax until the forces fell below 0.02 eV Å^−1^. The energy convergence criterion was set to 10^−5^ eV. The zero-point vibrational energy (ZPE) and entropy corrections were performed by vibrational frequency analysis. We applied a graphene supercell with the surface periodicity of 8 × 8 including 128 atoms as a basis to construct the M-pyrrolic N_4_ moieties. A vacuum region of 15 Å in the normal direction of the graphene plane was created to ensure negligible interaction between mirror images of the supercells.

## Supplementary information

Supplementary Information

## Data Availability

The data that support the findings of this study are available from the corresponding author upon reasonable request.
