# Supplementary material for: A general strategy for preparing pyrrolic-N4 type single-atom catalysts via pre-located isolated atoms
Source: Nat Commun. 2021 Nov 23;12:6806. doi: 10.1038/s41467-021-27143-5 (PMC8611002; doi:10.1038/s41467-021-27143-5)
Supplement: Supplementary file 1 — Supplementary Information [file 41467_2021_27143_MOESM1_ESM.pdf]

A General Strategy for Preparing Pyrrolic-N_4 Type Single-Atom Catalysts via Pre-located Isolated Atoms

Junjie Li[1, †], Ya-fei Jiang[2, †], Qi Wang[3, †], Cong-Qiao Xu[2], Duojie Wu[3], Mohammad Norouzi Banis[1], Keegan R. Adair[1], Kieran Doyle-Davis[1], Debora Motta Meira[4, 5], Y. Zou Finfrock[4, 5], Weihan Li[1], Lei Zhang[1], Tsun-Kong Sham[6], Ruying Li[1], Ning Chen[4, *], Meng Gu[3, *], Jun Li[2,7,*], Xueliang Sun[1, *]

[1]Department of Mechanical and Materials Engineering, University of Western Ontario, London, ON N6A 5B9, Canada.

[2]Department of Chemistry, Southern University of Science and Technology, Shenzhen 518055, China

[3]Department of Materials Science and Engineering, Southern University of Science and Technology, Shenzhen 518055, China

[4]Science Division, Canadian Light Source Inc., 44 Innovation Boulevard, Saskatoon, SK S7N 2V3, Canada.

[5]CLS @ APS, Advanced Photon Source, Argonne National Laboratory, Lemont, IL 60439, USA

[6]Department of Chemistry, University of Western Ontario, London, Ontario, Canada N6A 5B7, Canada

[7]Department of Chemistry and Key Laboratory of Organic Optoelectronics and Molecular Engineering of Ministry of Education, Tsinghua University, Beijing 100084, China.

Corresponding Email: ning.chen@lightsource.ca; gum@sustech.edu.cn; junli@tsinghua.edu.cn; xsun9@uwo.ca

[†]These authors contributed equally to this work

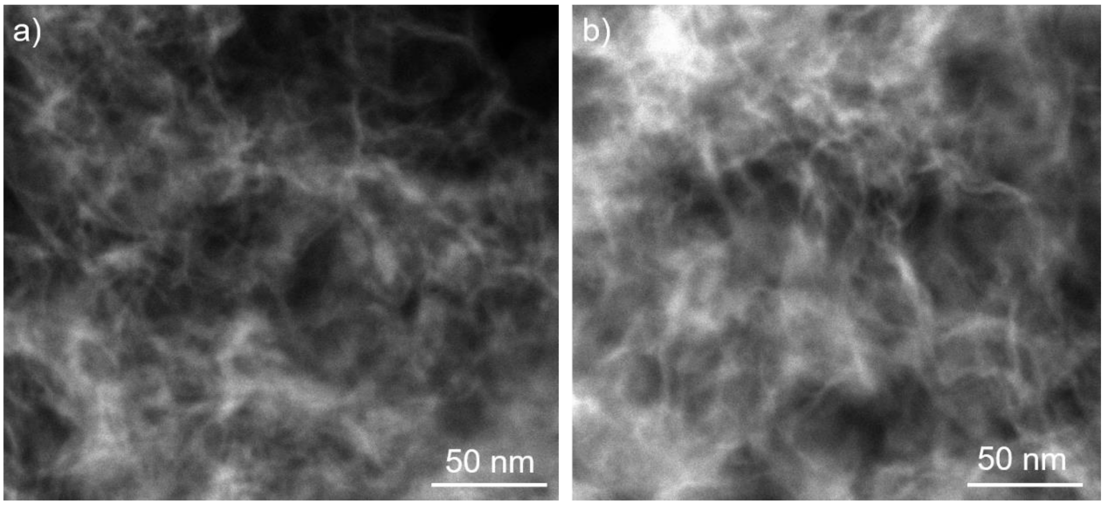

Supplementary Fig. 1 HAADF-STEM images of NCNS at different locations (a and b).

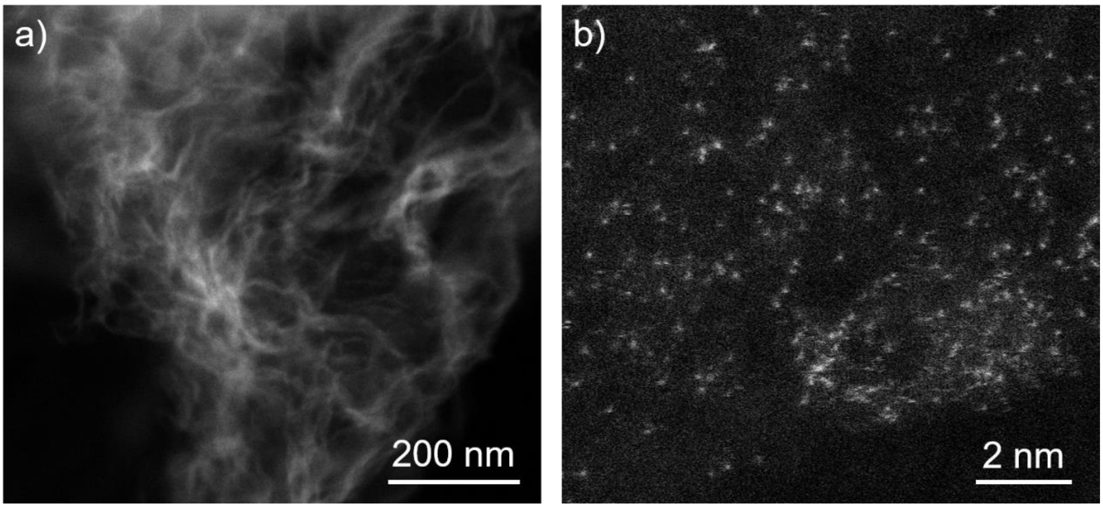

Supplementary Fig. 2 HAADF-STEM images of Pt_1/NCNS at low (a) and high (b) magnification.

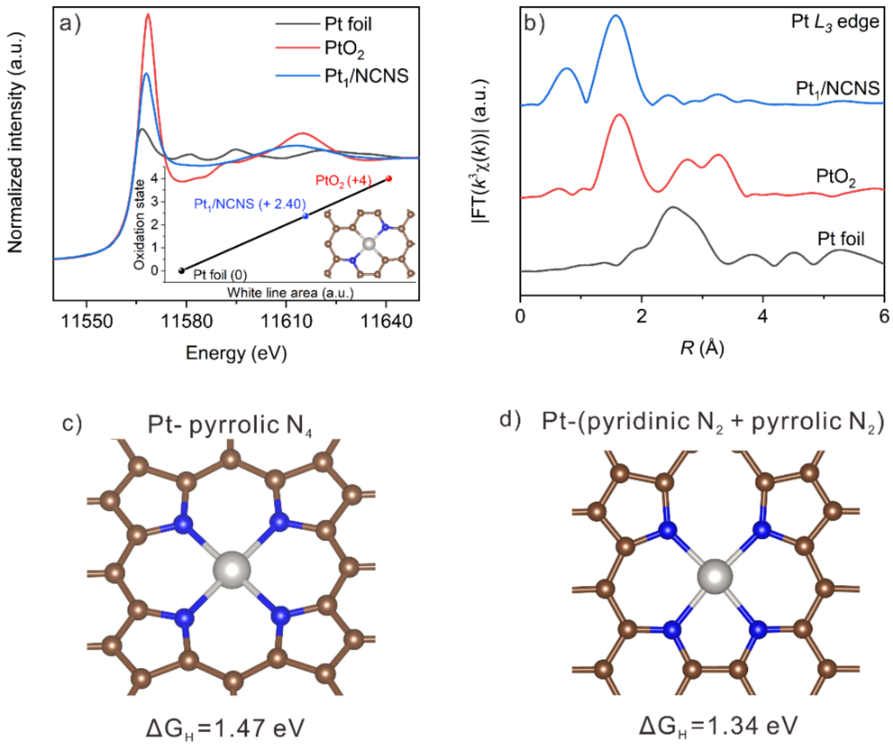

Supplementary Fig. 3 (a) XANES and (b) EXAFS of Pt_1/NCNS, as well as Pt foil and PtO_2 reference at Pt L_3 edge. Inset: averaged oxidation state of Pt on Pt_1/NCNS based on the white line area. (c and d) DFT optimized possible structures and calculated Gibbs free energy profiles of HER at the equilibrium potential of Pt SAC at T = 298.15 K and P = 1atm.

Note:

The Pt L_3 edge XANES result of Pt_1/NCNS is distinct from the Pt foil, it shows a very intense high white line (WL) at higher photon energy compared to that of Pt foil, indicating Pt is at a high oxidization state with a significant increase in 5*d* hole counts compared to Pt metal (**Supplementary Fig. 3a**). It is established that the area under the WL curve is proportional to the total unoccupied state of Pt 5*d* orbitals which also can reflect the oxidation state of Pt_1 atoms[1]. After quantitative analysis of the WL area, we found that the average oxidation state of Pt_1 atom is around +2.4, indicating the Pt could bond with two N atoms (**Supplementary Fig. 3a**)[2].

We have built two possible structures of Pt SACs with pyrrole N sites, as shown in **Supplementary Figs. 3c and d**. However, both of them show high Gibbs free energy barrier (ΔG_H), indicating the poor HER activity, which differs from the HER performance of Pt_1/NCNS[3]. Therefore, we conclude the Pt_1 atom prefers to coordinate pyridine N rather than pyrrole N sites.

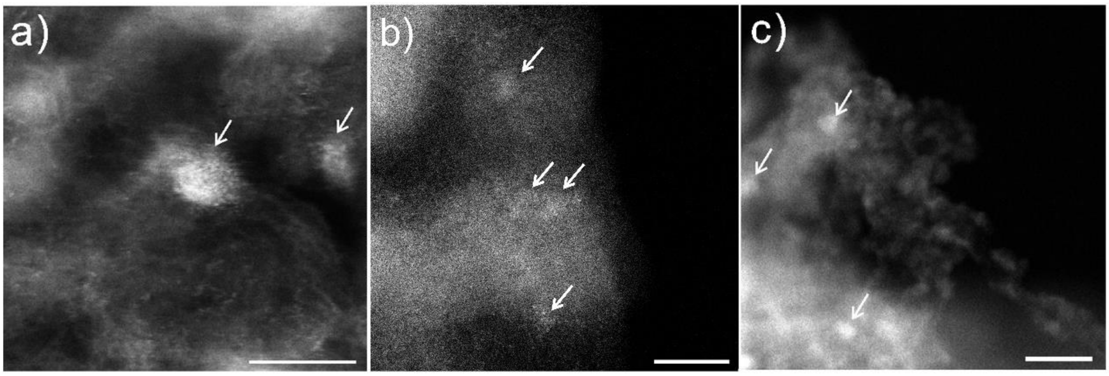

Supplementary Fig. 4 HAADF-STEM images of Fe (a), Co (b), Ni-NPs/NCNS (c). Scale bar: 5 nm. The white arrows highlight the clusters/NPs.

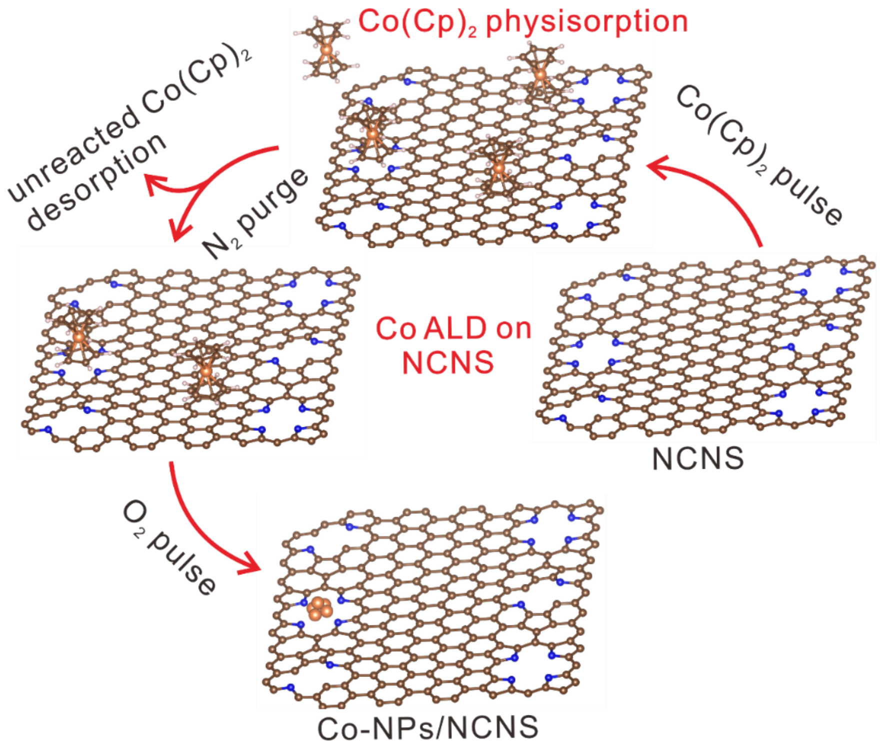

Supplementary Fig. 5 Illustration of Co ALD process on NCNS. The white, blue, brown, and orange spheres represent H, N, C, and Co, respectively.

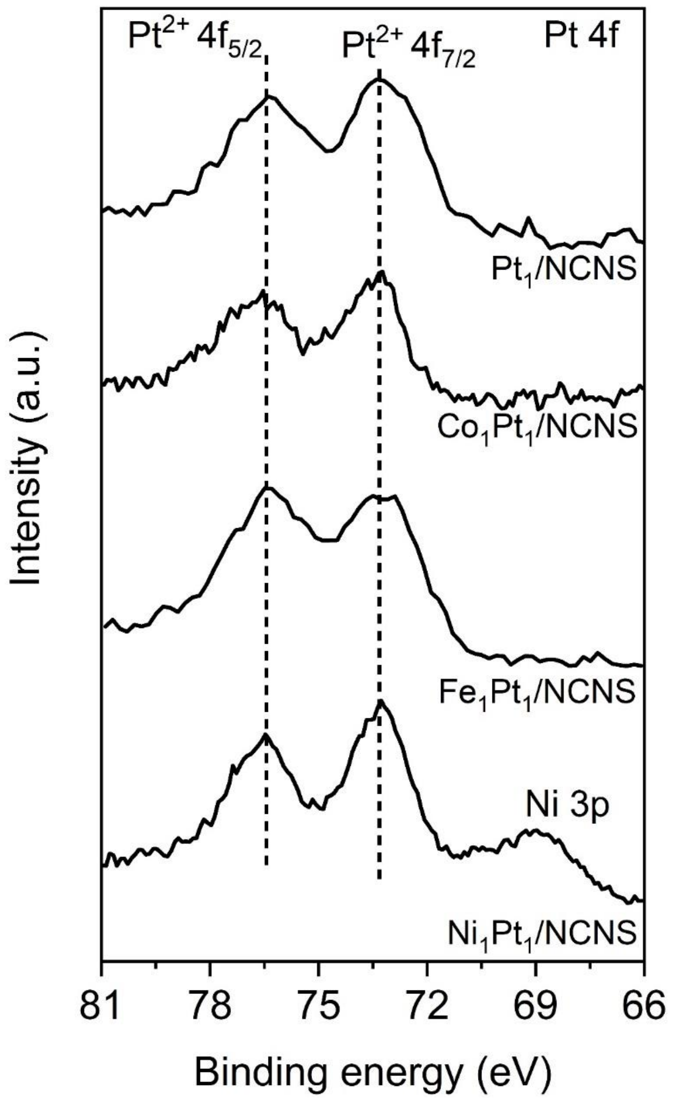

Supplementary Fig. 6 XPS results of Pt_1/NCNS, Co_1Pt_1/NCNS, Fe_1Pt_1/NCNS, and Ni_1Pt_1/NCNS at Pt 4f region.

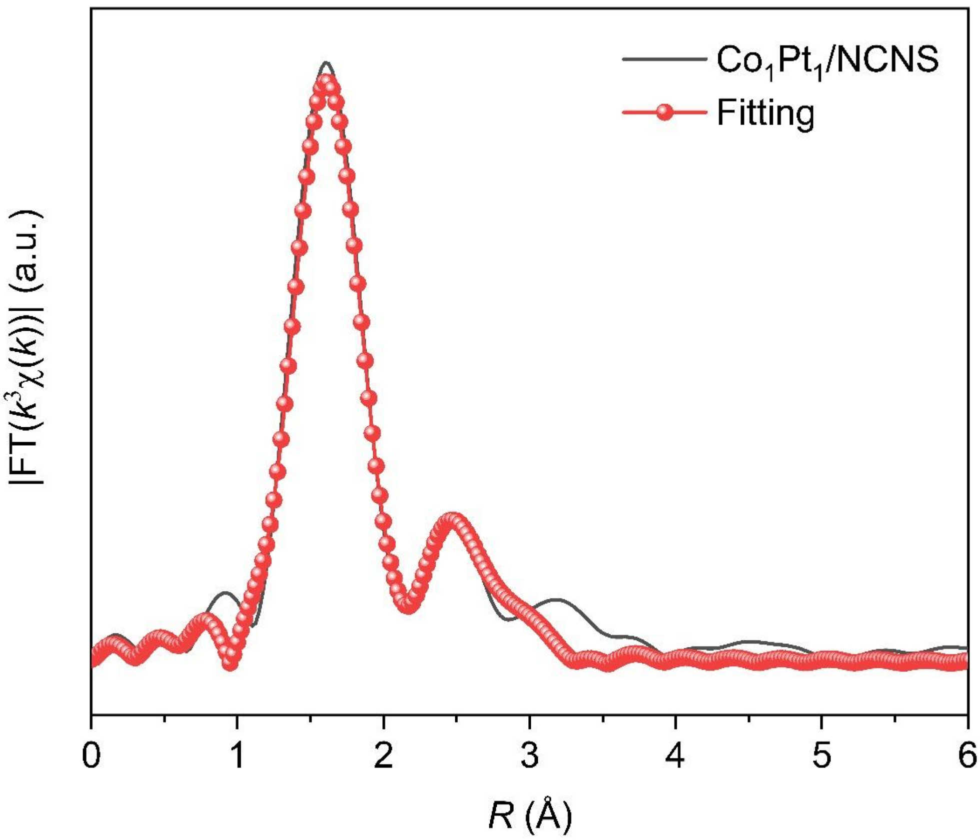

Supplementary Fig. 7 k^3-weighted FT spectrum in R space fitting for Co_1Pt_1/NCNS at Pt L_3 edge.

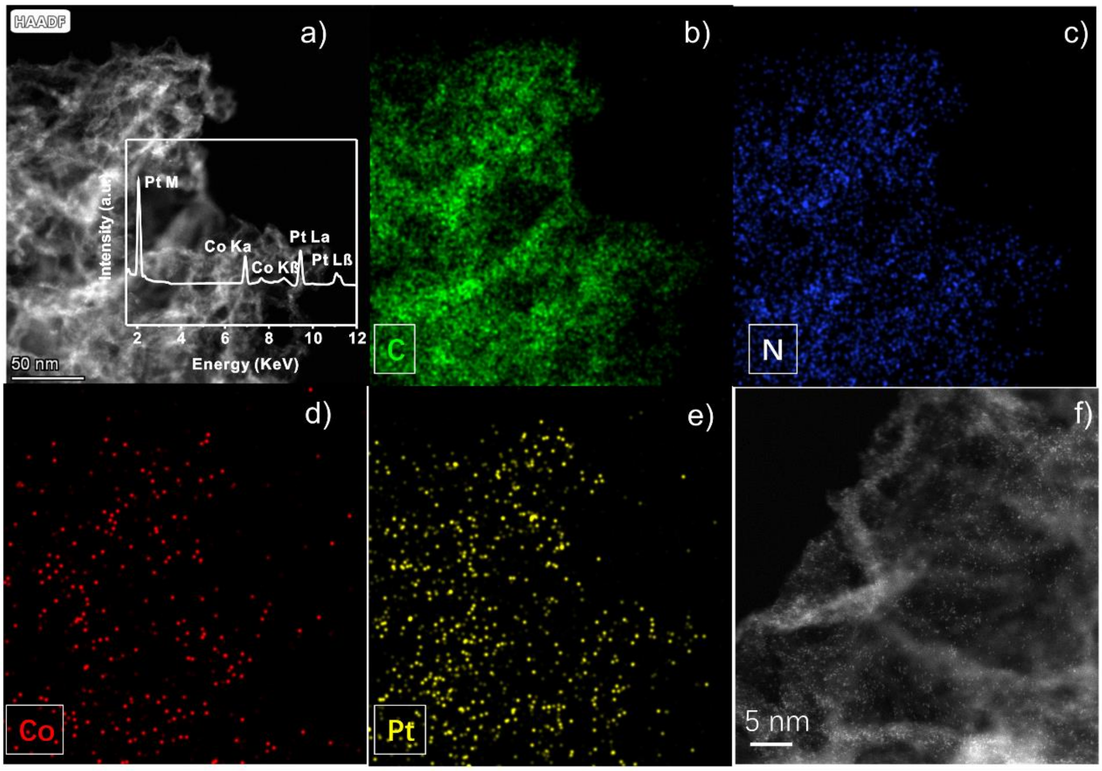

Supplementary Fig. 8 (a) STEM image and EDX elemental analysis. (b to e) EDX mapping. (f) high magnification HAADF-STEM image of Co_1Pt_1/NCNS.

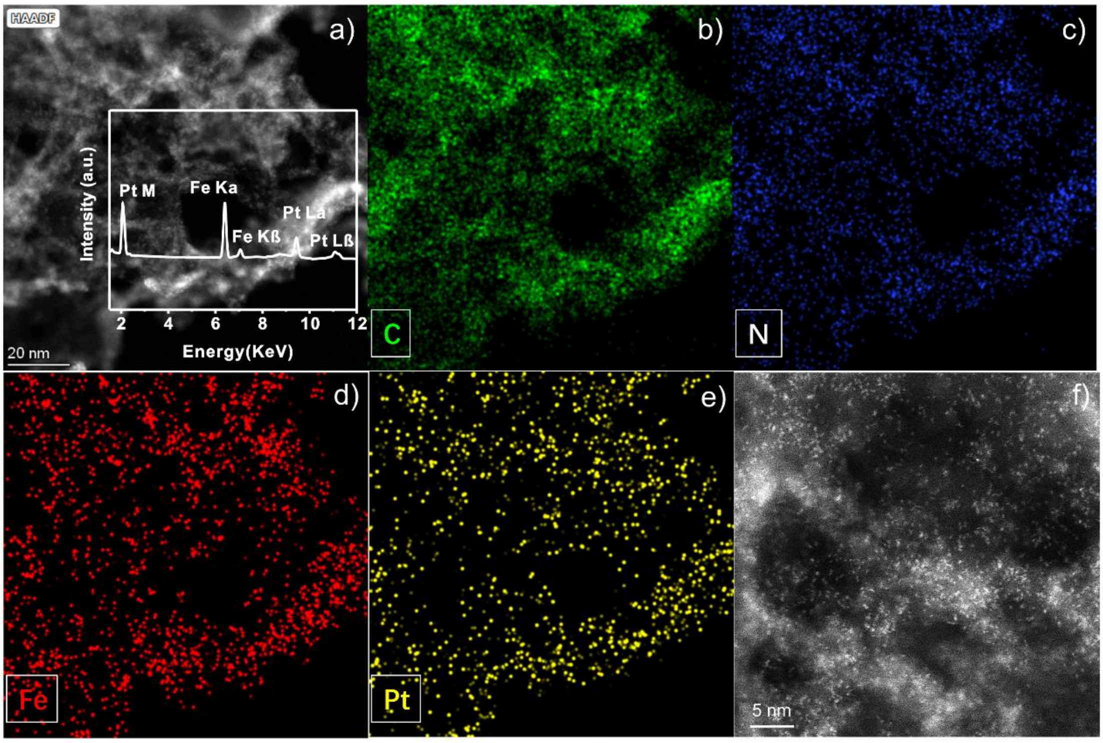

Supplementary Fig. 9 (a) STEM image and EDX elemental analysis. (b to e) EDX mapping. (f) high magnification HAADF-STEM image of Fe_1Pt_1/NCNS.

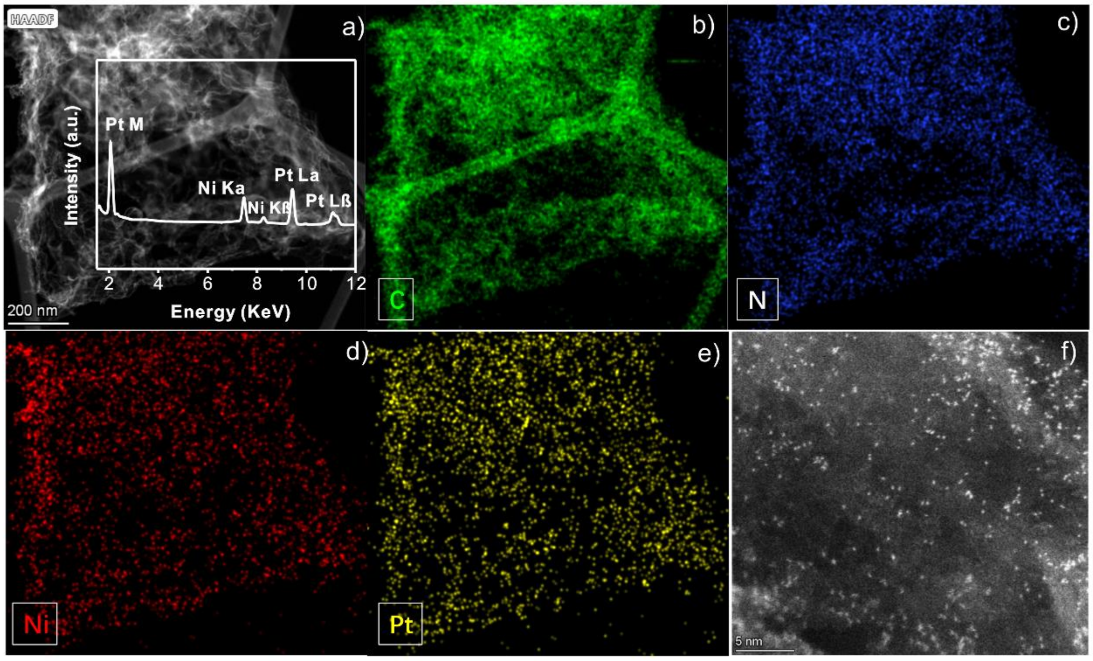

Supplementary Fig. 10 (a) STEM image and EDX elemental analysis. (b to e) EDX mapping. (f) high magnification HAADF-STEM image of Ni_1Pt_1/NCNS.

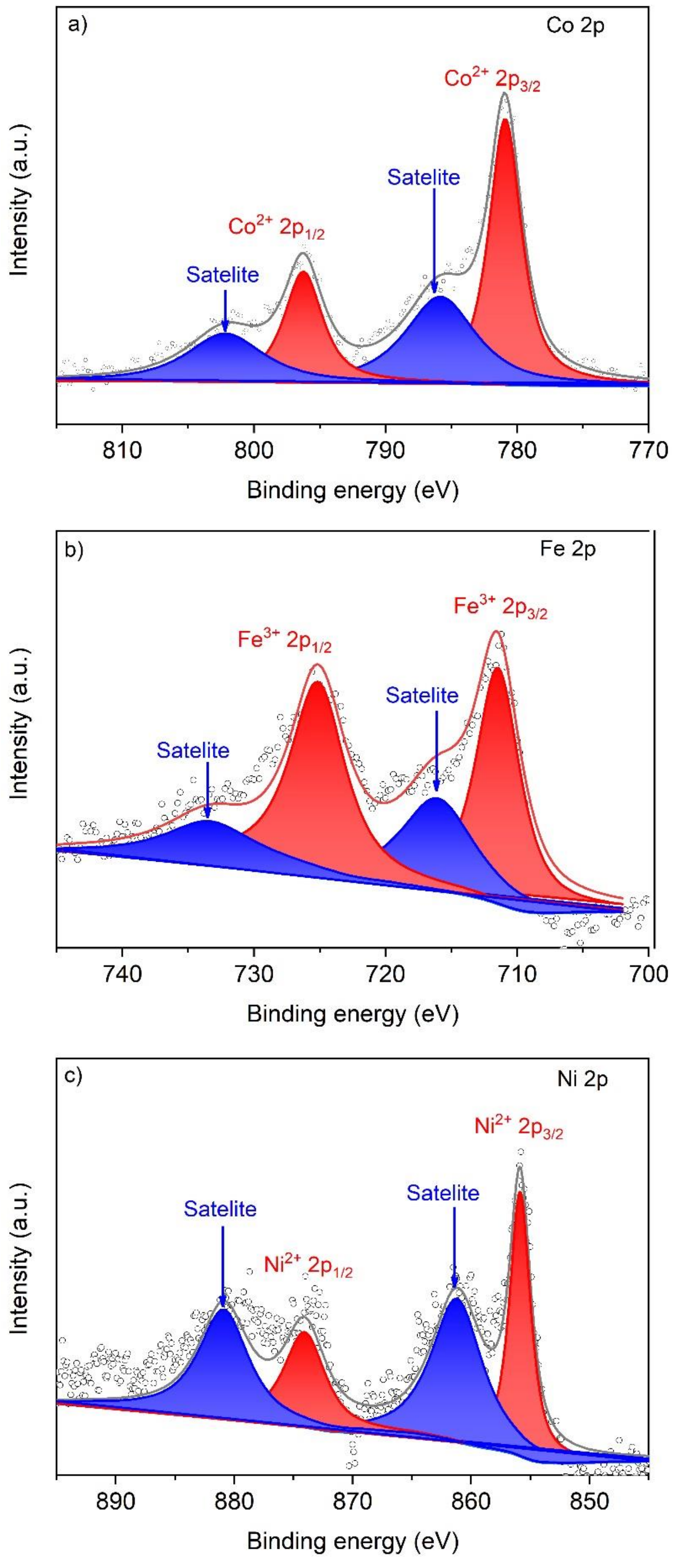

Supplementary Fig. 11 XPS results of Co_1Pt_1/NCNS at Co 2p region (a), Fe_1Pt_1/NCNS at Fe 2p region (b), and Ni_1Pt_1/NCNS at Ni 2p region (c).

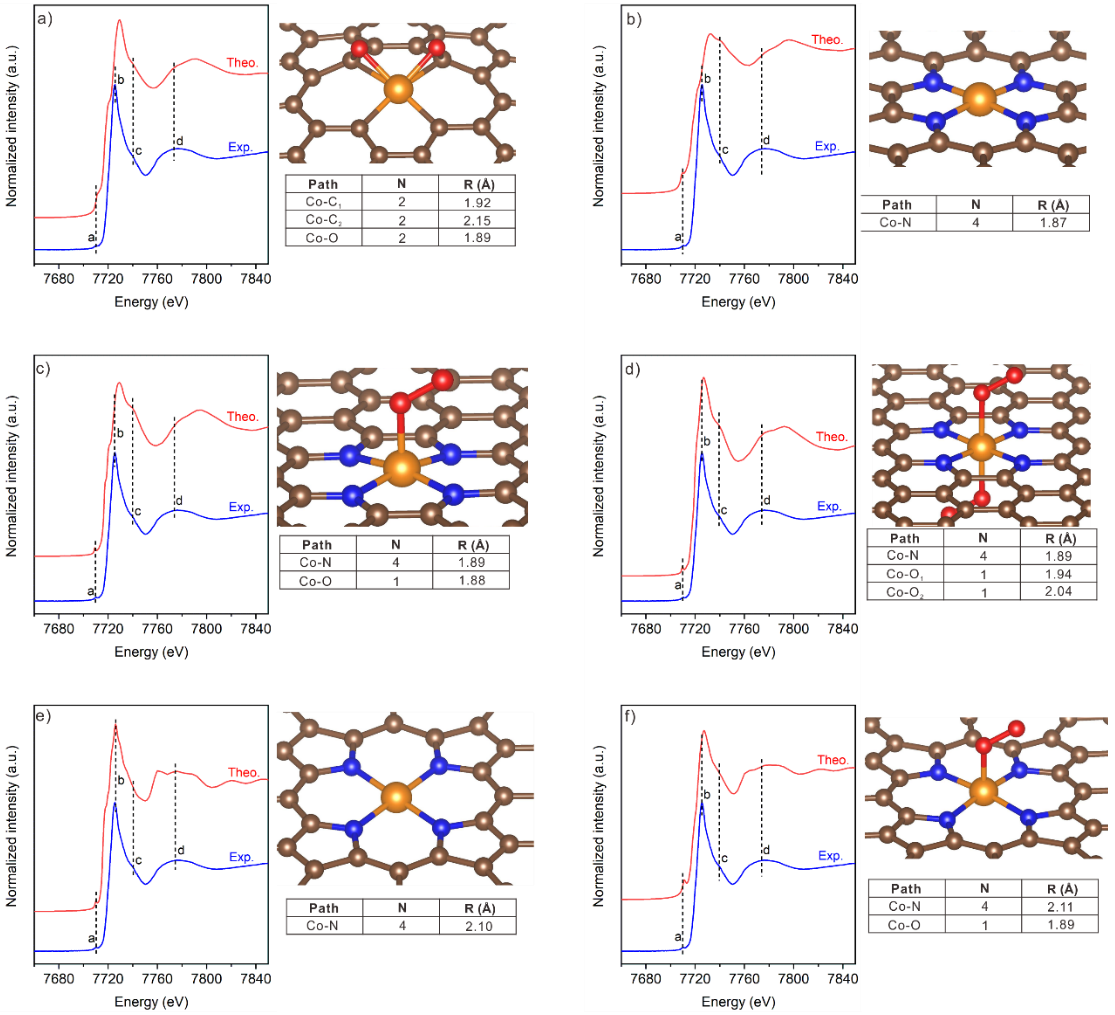

Supplementary Fig. 12 (a to f) Comparison between the experimental Co *K*-edge XANES spectrum of Co_1Pt_1/NCNS and computational spectra based on the optimized structures. The white, blue, brown, red, and orange spheres represent H, N, C, O, and Co, respectively.

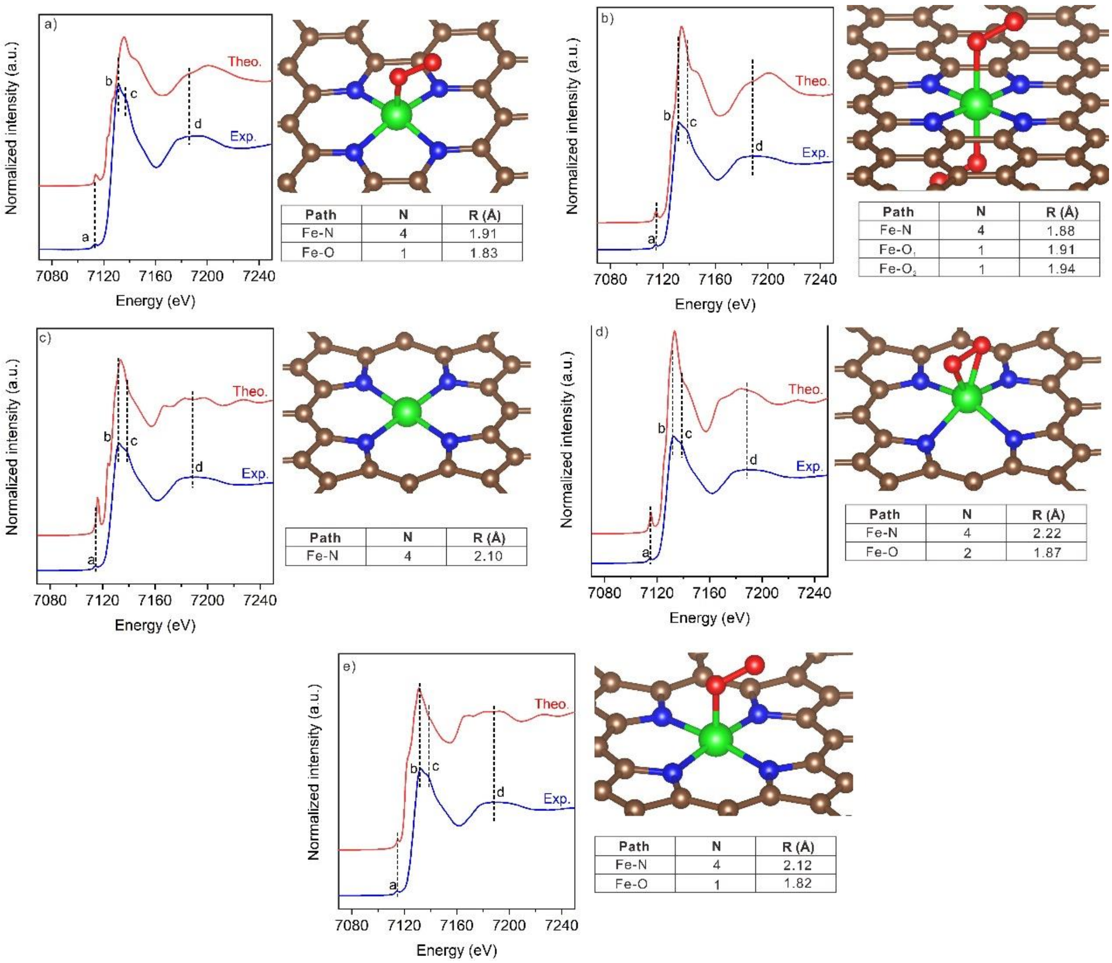

Supplementary Fig. 13 (a to e) Comparison between the experimental Fe *K*-edge XANES spectrum of Fe_1Pt_1/NCNS and computational spectra based on the optimized structures. The white, blue, brown, red, and green spheres represent H, N, C, O, and Fe, respectively.

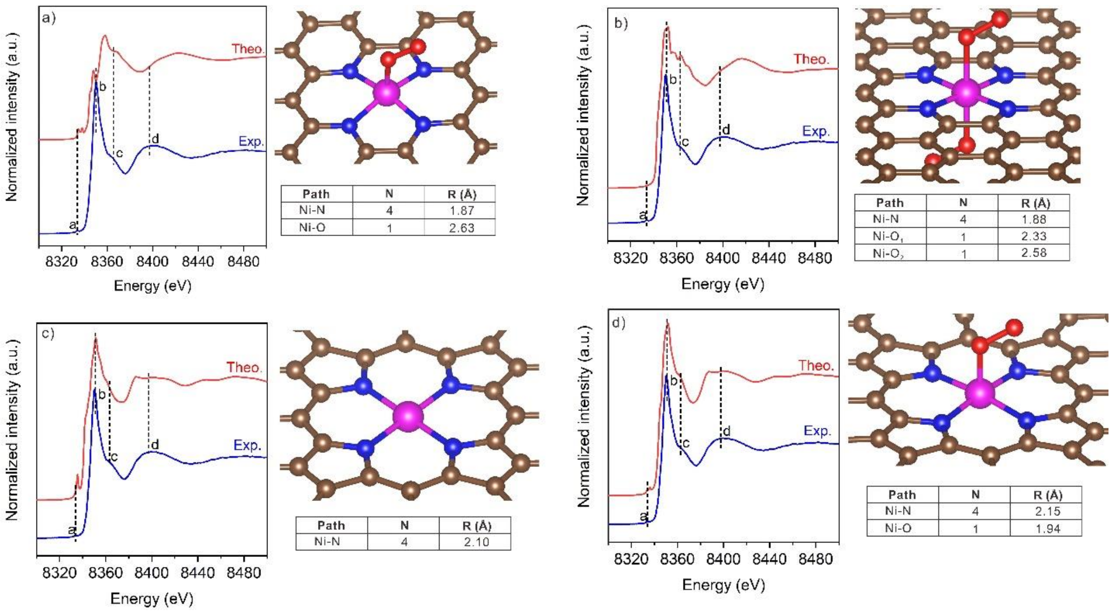

Supplementary Fig. 14 (a to d) Comparison between the experimental Ni *K*-edge XANES spectrum of Ni_1Pt_1/NCNS and computational spectra based on the optimized structures. The white, blue, brown, red, and pink spheres represent H, N, C, O, and Ni, respectively.

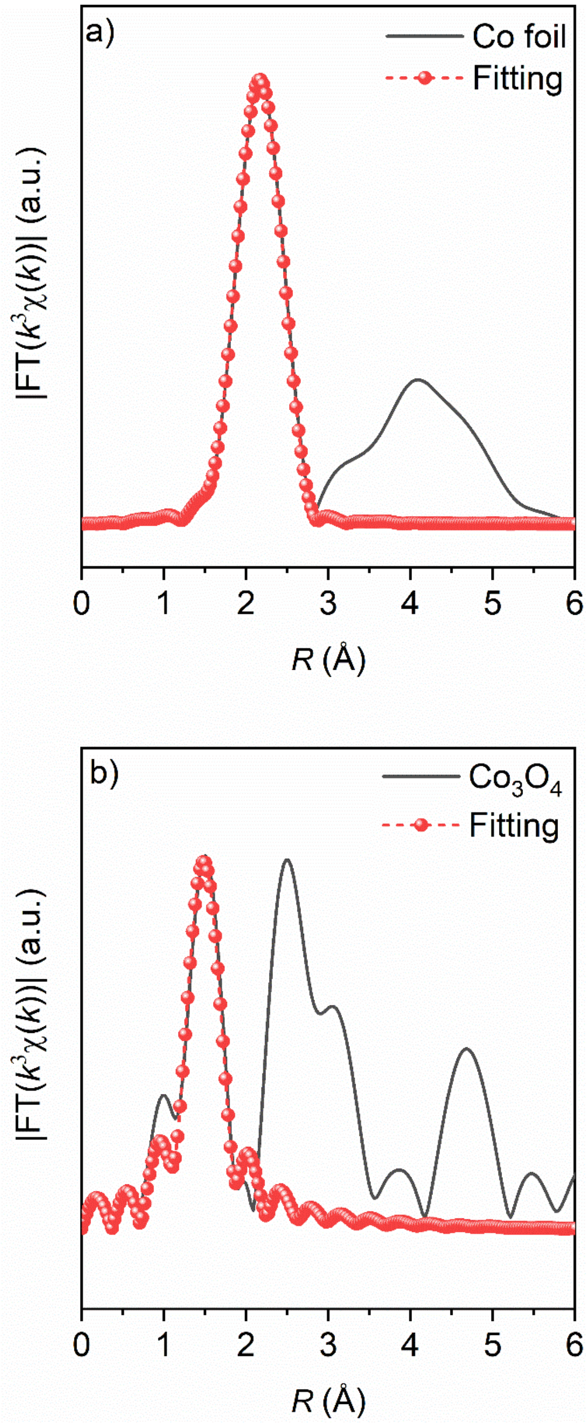

Supplementary Fig. 15 k^3-weighted FT spectrum in R space fitting for Co foil (a) and Co_3O_4 (b) at Co *K* edge.

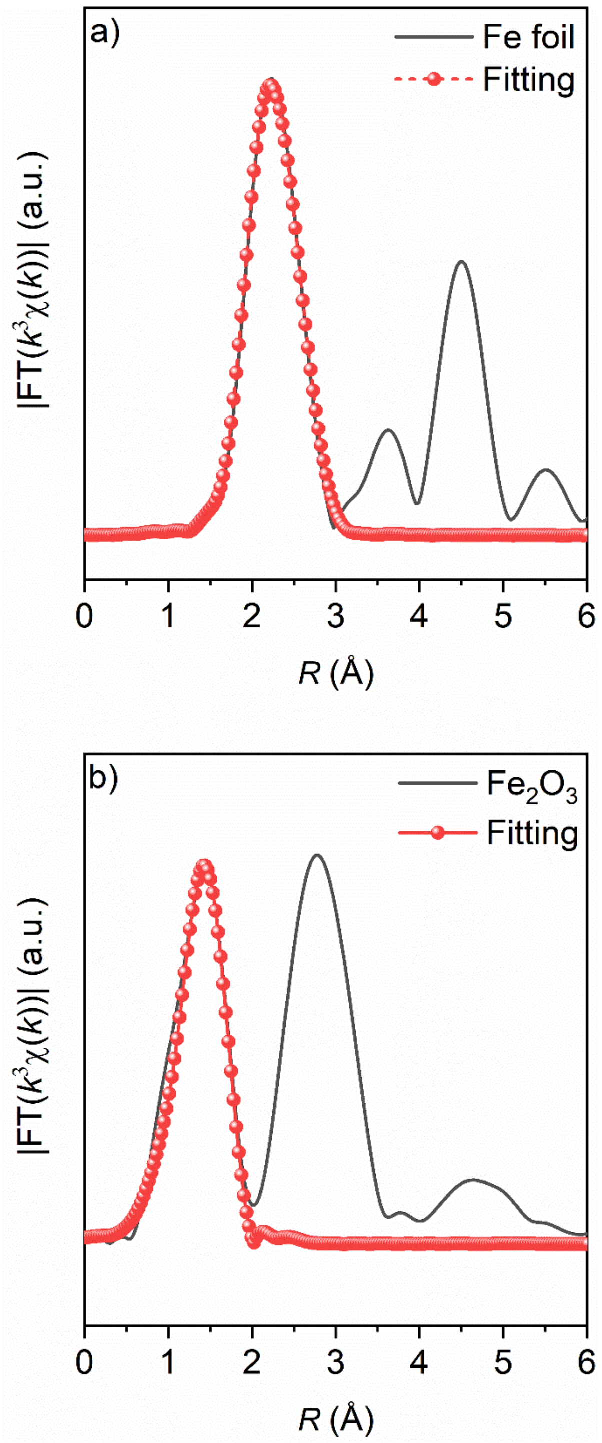

Supplementary Fig. 16 k^3-weighted FT spectrum in R space fitting for Fe foil (a) and Fe_2O_3 (b) at Fe *K* edge.

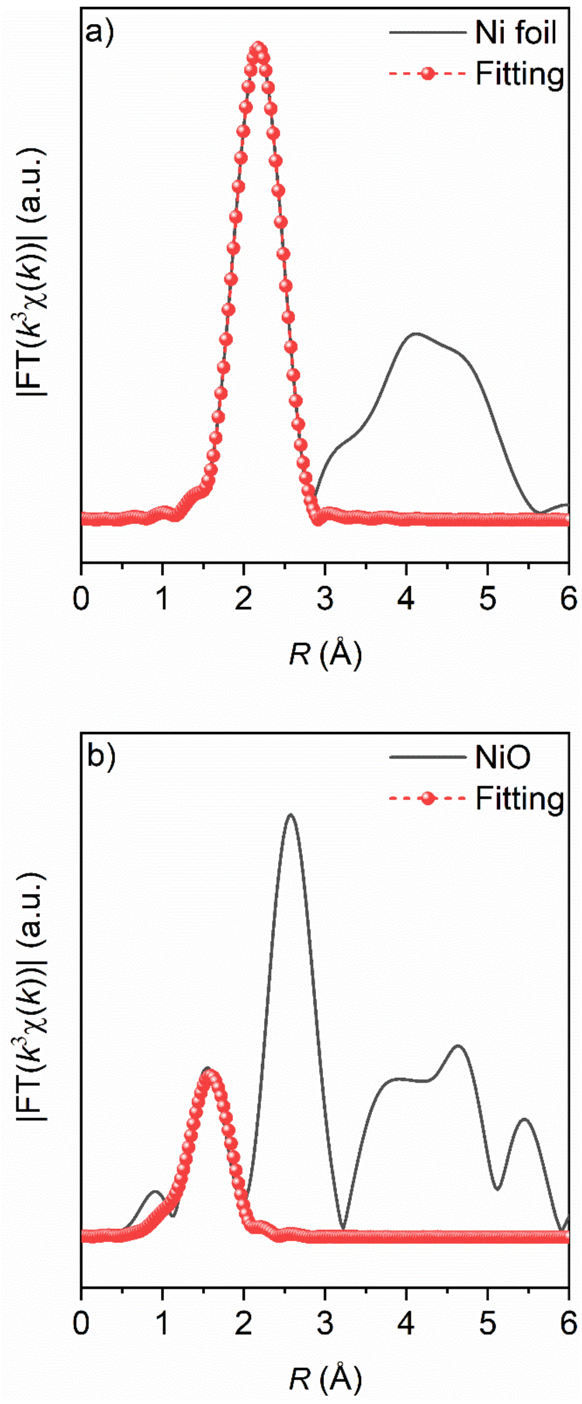

Supplementary Fig. 17 k^3-weighted FT spectrum in R space fitting for Ni foil (a) and NiO (b) at Ni *K* edge.

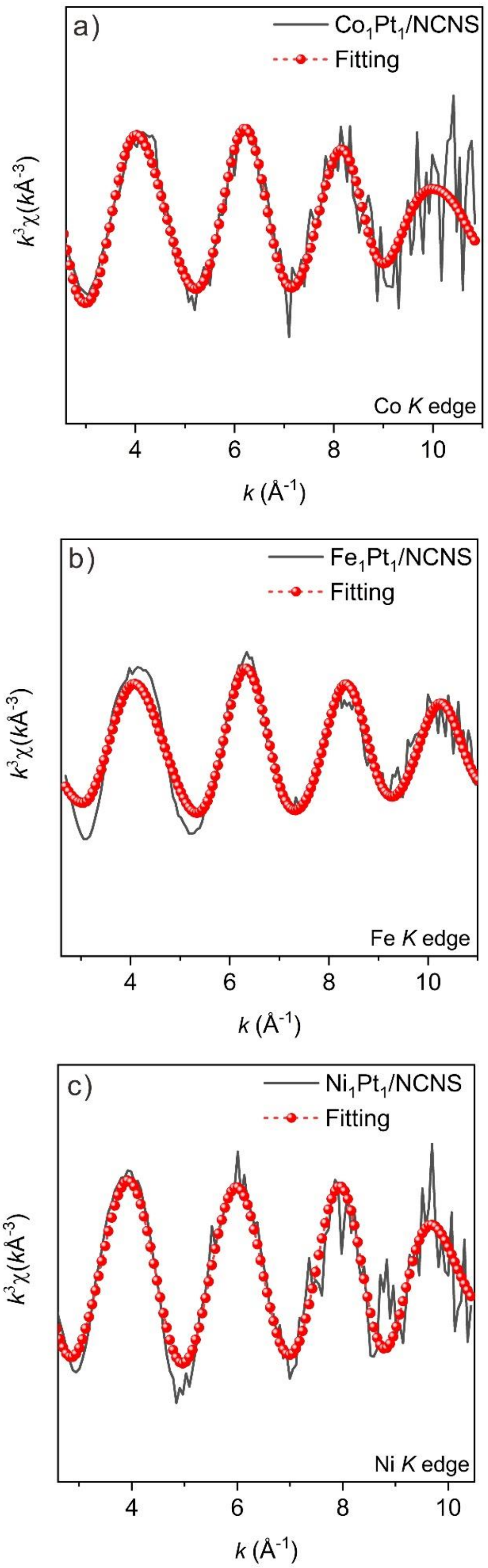

Supplementary Fig. 18 k^3-weighted FT spectrum in k space fitting for M_1Pt_1/NCNS at Co (a), Fe (b), and Ni (c) *K* edges.

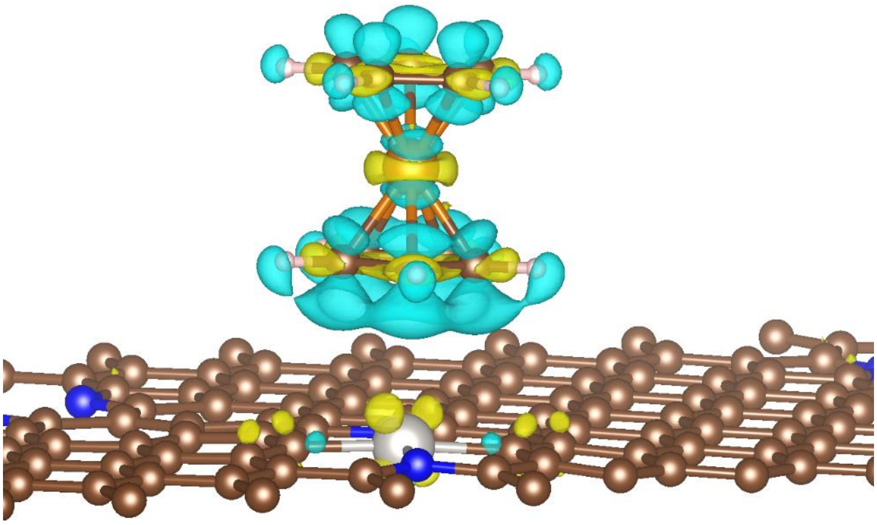

Supplementary Fig. 19 Calculated charge density difference for $Co(Cp)_2$ adsorption on the Pt_1/NCNS. Yellow and cyan iso-surface represent electron accumulation and electron depletion, respectively. The white, blue, brown, orange, and silver spheres represent H, N, C, Co, and Pt, respectively.

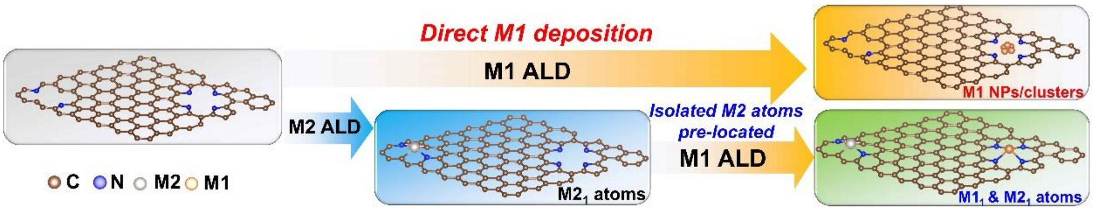

Supplementary Fig. 20 Illustration of the synthesis process to achieve SACs using pre-located isolated atoms.

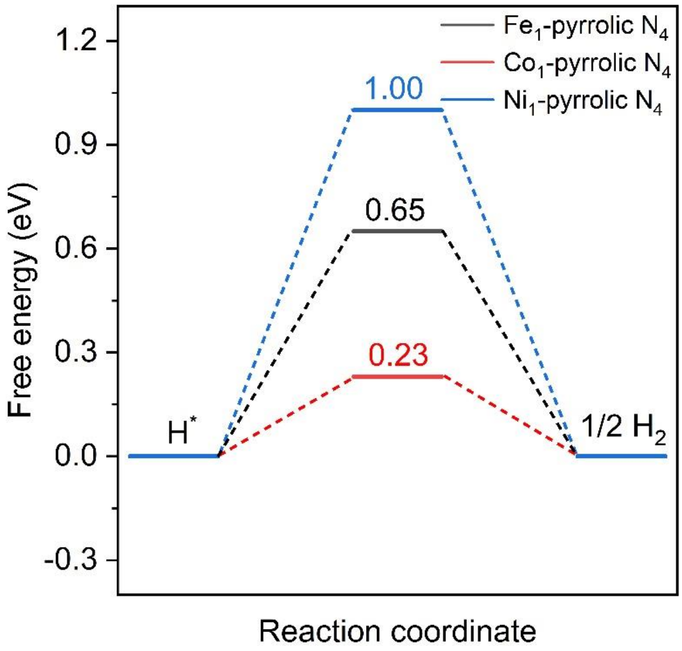

Supplementary Fig. 21 Calculated Gibbs free energy profiles of HER at the equilibrium potential on M_1-pyrrolic N_4 sites at T = 298.15 K and P = 1 atm.

.

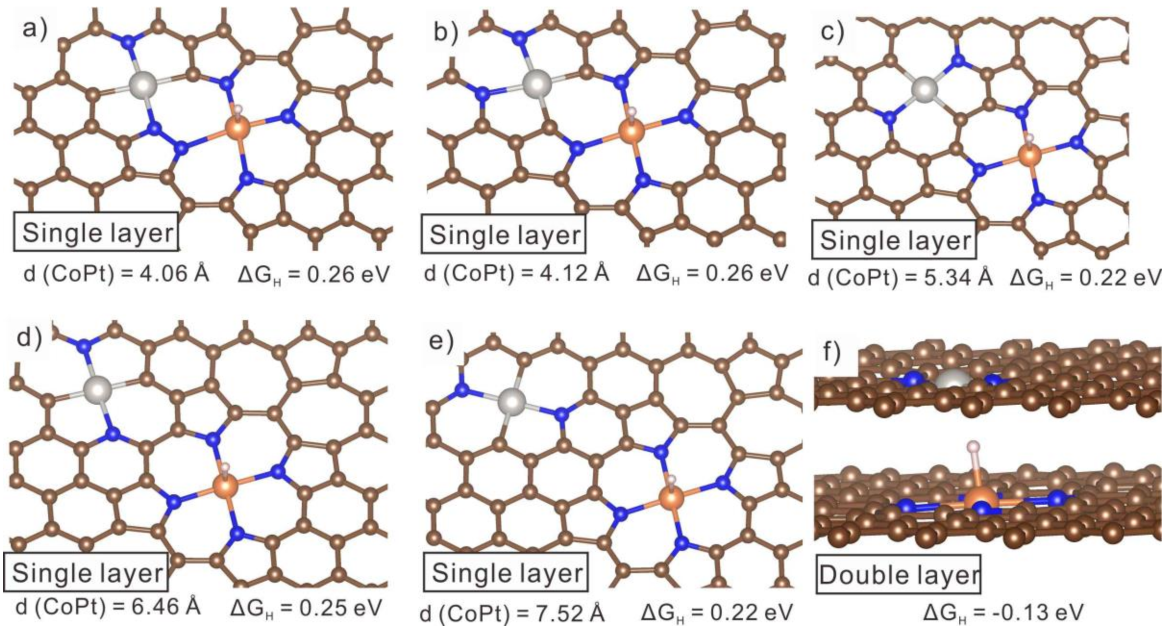

Supplementary Fig. 22 DFT optimized structures and calculated Gibbs free energy profiles of HER at the equilibrium potential of single-layered (a to e) and double-layered (f) Co_1Pt_1/NCNS at T = 298.15 K and P = 1 atm.

Note:

As the Co_1Pt_1/NCNS exhibits the best catalytic activity, we have taken it as a typical example for the further theoretical understanding of the interaction of Co_1 and Pt_1 in the HER.

1) Considering the Co_1Pt_1/NCNS is in a single-layered structure, we have adjusted the distances between Co_1 and Pt_1 single atoms to investigate the different catalytic activity of Co_1 single atoms in HER (**Supplementary Figs. 22a to e**). However, the hydrogen adsorption free energies (ΔG_H) at T = 298.15K and P = 1atm are almost the same, despite different distances between Co_1 and Pt_1 single atoms, indicating the interaction of Pt_1 and Co_1 atoms is less pronounced in the single-layered structure.
2) According to the literature, the interaction between two single atoms in the double-layered structure could promote catalytic performance[4,5]. We further consider the bilayer structure in our system and found the interactions between Co_1 and Pt_1 in each layer are strongly enhanced, with the bond distance of Co-Pt decreased to 2.91 Å. The ΔG_H of Co_1 is -0.13 eV on the double-layered model, which is much lower than that in the single-layer structure (0.23 eV) (**Supplementary Fig. 22f**).

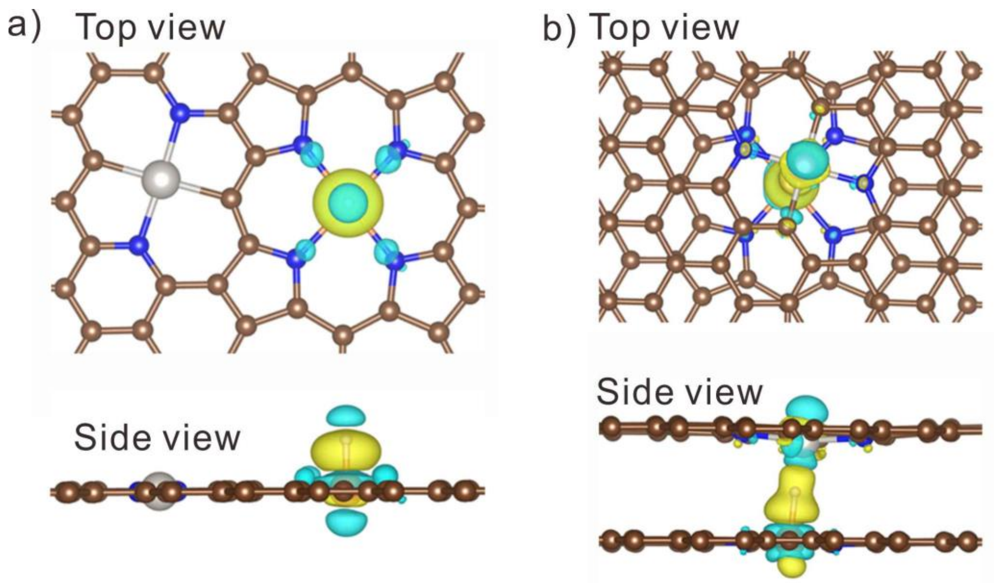

Supplementary Fig. 23 Top and side view of the calculated charge distribution of single (a) and double (b)-layered Co_1Pt_1/NCNS. Yellow and cyan iso-surface represent electron accumulation and electron depletion, respectively. The white, blue, brown, orange, and silver spheres represent H, N, C, Co, and Pt, respectively.

Note:
We have compared the charge distributions of single/double layered Co_1Pt_1/NCNS (**Supplementary Figs. 23a and b**). As shown in **Supplementary Fig. 23**, the charge polarization on double-layered Co_1Pt_1/NCNS shows obvious differences between single and double-layered models. Differing from the single-layered structure, the electron accumulation is strongly enhanced by the interlayer on the double-layered Co_1Pt_1/NCNS[6]. As a result, the adsorption of protons is facilitated (**Supplementary Fig. 22f**). Therefore, the Co_1Pt_1/NCNS shows better performance than the Pt_1/NCNS in HER could be likely due to the extra contribution of Co_1 pyrrolic-N_4 sites in both single/double-layered structures.

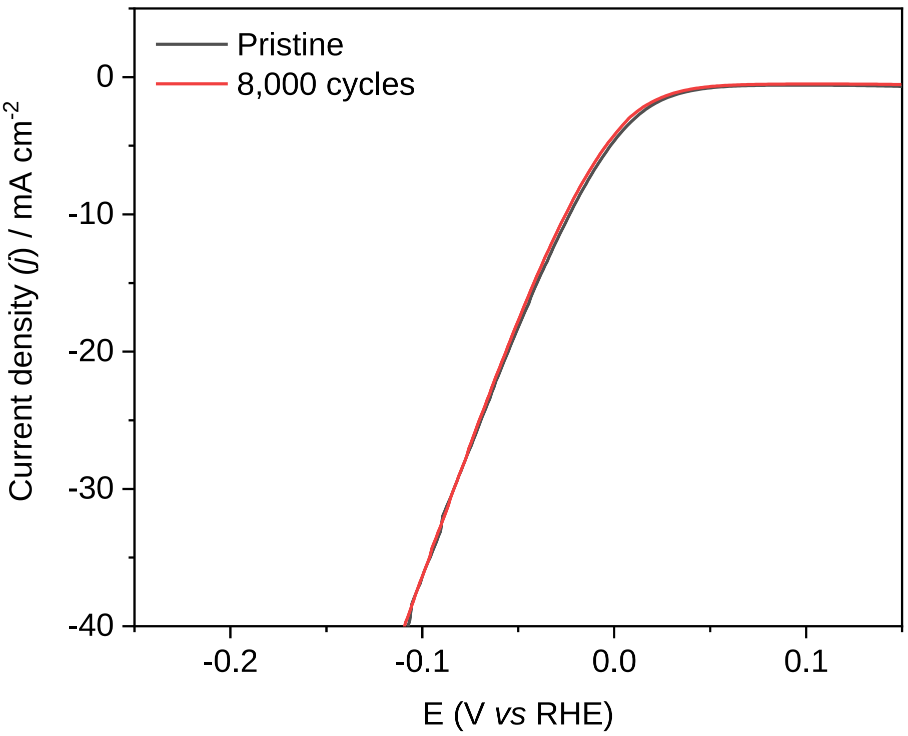

Supplementary Fig. 24 The durability test of Co_1Pt_1/NCNS without IR correction at the sweep voltage between -0.1 and 0.4 V at a scan rate of 0.1 V s^{-1} in 0.5 M H_2SO_4.

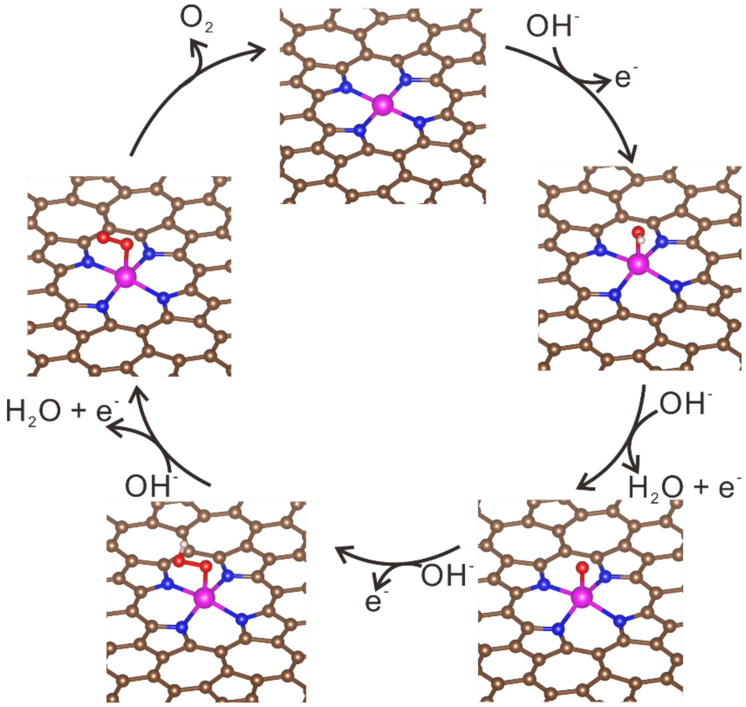

Supplementary Fig. 25 Computationally proposed reaction scheme for OER at alkaline condition on Ni_1-pyrrolic N_4 site. The brown, blue, red, and pink spheres represent C, N, O, and Ni, respectively.

Note:

The OER occurs at alkaline conditions via the following steps:

$$* + 4OH^- \rightarrow *OH + 3OH^- + e^-$$

$$*OH + 3OH^- + e^- \rightarrow *O + 2OH^- + H_2O + 2e^-$$

$$*O + 2OH^- + H_2O + 2e^- \rightarrow *OOH + OH^- + H_2O + 3e^-$$

$$*OOH + OH^- + H_2O + 3e^- \rightarrow *OO + 2H_2O + 4e^-$$

$$*OO + 2H_2O + 4e^- \rightarrow * + O_2 + 2H_2O + 4e^-$$

where * denotes the active site.

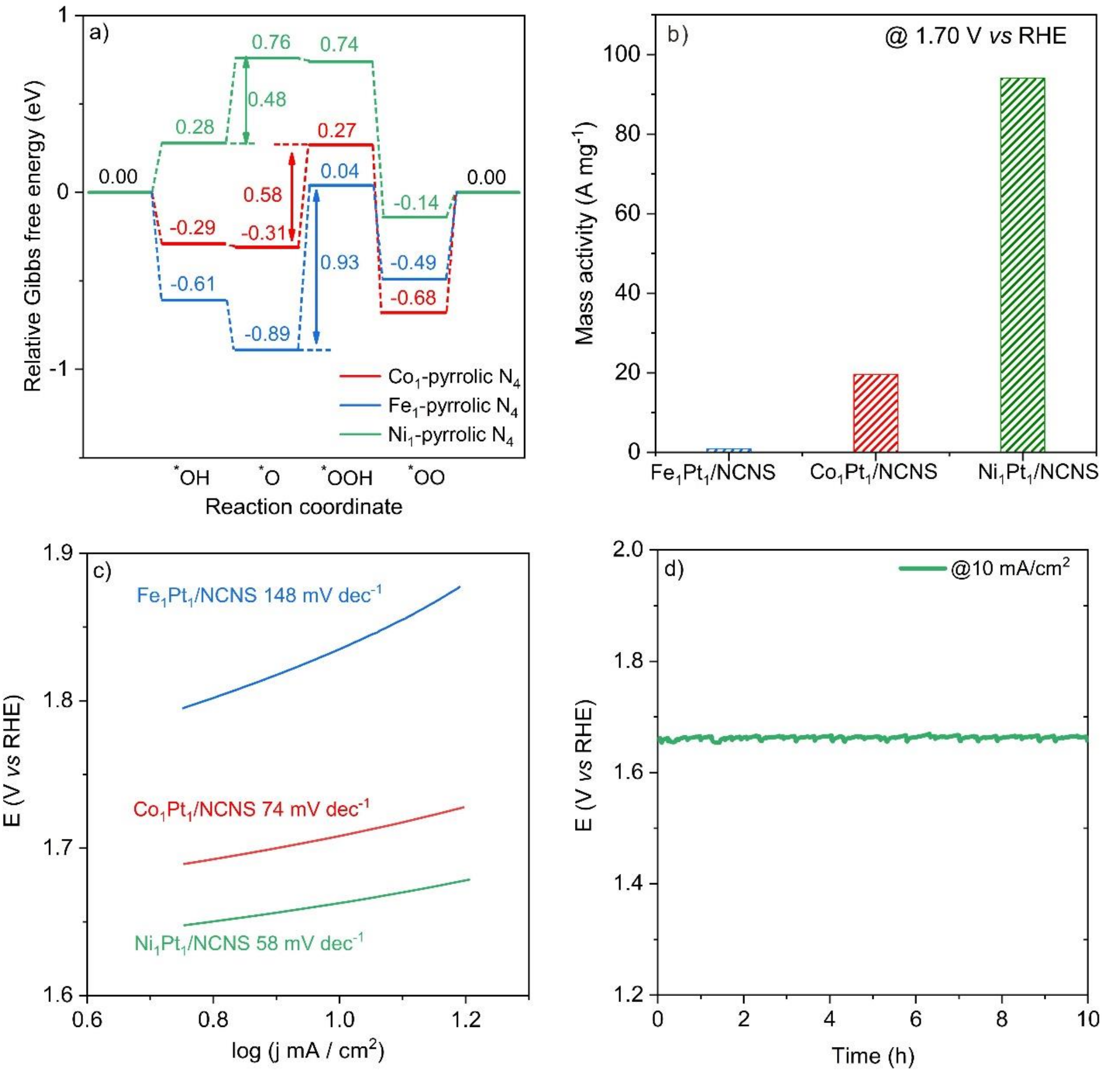

Supplementary Fig. 26 Theoretically predicted activity of OER and electrochemical performance of M_1Pt_1/NCNS (M = Co, Fe, and Ni). (a) Relative Gibbs free energy for M_1Pt_1/NCNS for OER at 1.23 V at T = 298.15 K and P = 1 atm. (b) Mass activity of Fe_1Pt_1/NCNS, Co_1Pt_1/NCNS and Ni_1Pt_1/NCNS at 1.70 V (*vs* RHE). (c) Tafel plots of M_1Pt_1/NCNS catalysts. (d) The durability test of Ni_1Pt_1/NCNS at a constant current density of 10 mA/cm^2.

Note:

Although it has been reported in the literature that the pyridinic-N_4 type non-noble metal SACs exhibited high catalytic performance in OER, the investigation of pyrrolic-N_4 type SACs is rarely studied[7]. Note that the axial O_2 molecules are not considered in the theoretical calculation because of the O_2 evolution process. According to the literature, the *d*-band center near the Fermi level is linked with the adsorption of reactants[8]. As shown in **Fig. 6a and Supplementary Table 4**, the *d*-band center of

metal atom in M_1Pt_1/NCNS increases from Ni to Fe, indicating there would be much stronger adsorption of oxygen species on Fe_1 atom than that on Ni_1 and Co_1 atoms. Based on previous work [9], we theoretically investigated the OER mechanism on M_1-pyrrolic N_4 (M = Co, Fe, and Ni) catalysts by considering the adsorption of *OH, *O, *OOH, and *OO intermediates (**Supplementary Fig. 25**), with the Gibbs free energy diagram calculated at T = 298.15K and P = 1atm shown in **Supplementary Fig. 26a**. It is found that the rate-determining step (RDS) is the oxidation of $*OH^*$ to *O with an energy barrier of 0.48 eV for Ni_1Pt_1/NCNS. However, the formation of *OOH becomes the RDS for Co_1Pt_1/NCNS and Fe_1Pt_1/NCNS, which possesses a higher energy barrier of 0.58 and 0.93 eV, respectively. Thus, we can predict that the OER activity on these three SACs would follow the order of Ni_1Pt_1/NCNS > Co_1Pt_1/NCNS > Fe_1Pt_1/NCNS.

The mass activity of Fe_1, Co_1, Ni_1Pt_1/NCNS at 1.70 V (*vs* RHE) is based on the loading of Fe, Co, and Ni (**Supplementary Fig. 26b**). The Ni_1Pt_1/NCNS exhibits the highest mass activity of 94.1 A/mg_{Ni}, which is more than four times higher than that of Co_1Pt_1/NCNS (19.6 A/mg_{Co}). The Tafel plots further demonstrate the kinetic advantage of Ni_1Pt_1/NCNS, with a lower Tafel slope (58 mV/dec) than Co_1Pt_1/NCNS (74 mV/dec) and Fe_1Pt_1/NCNS (148 mV/dec) (**Supplementary Fig. 26c**). More importantly, the Ni_1Pt_1/NCNS exhibits significant stability, without showing an obvious activity decrease after 10 h at a current density of 10 mA/cm^2 in durability testing (**Supplementary Fig. 26d**).

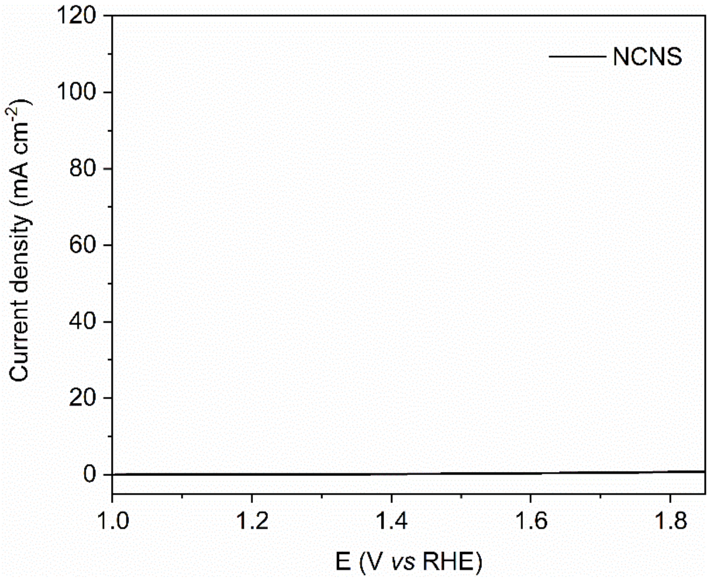

Supplementary Fig. 27 OER LSV curves NCNS under 1 M KOH at a scan rate of 5 mV/s.

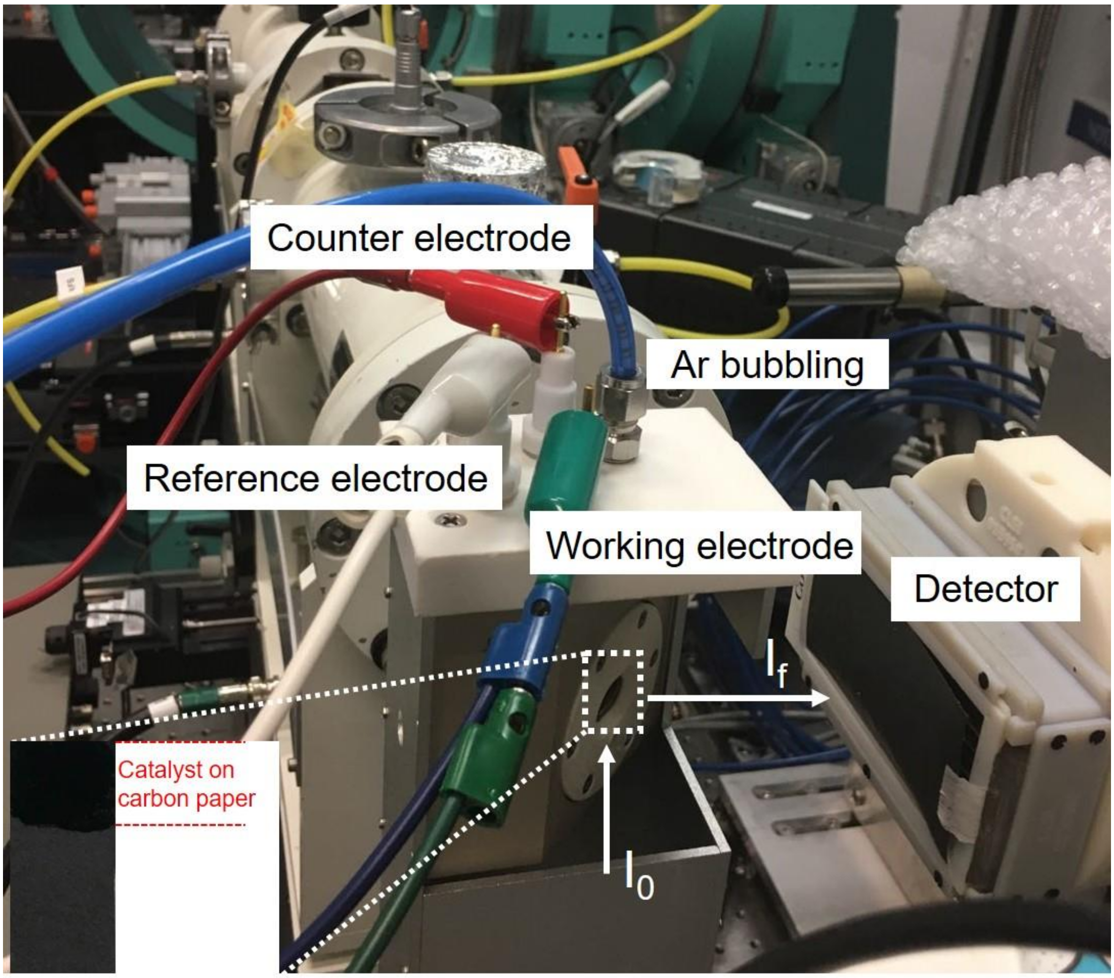

Supplementary Fig. 28 Illustration of the electrochemical cell used for *operando* XAS measurements.

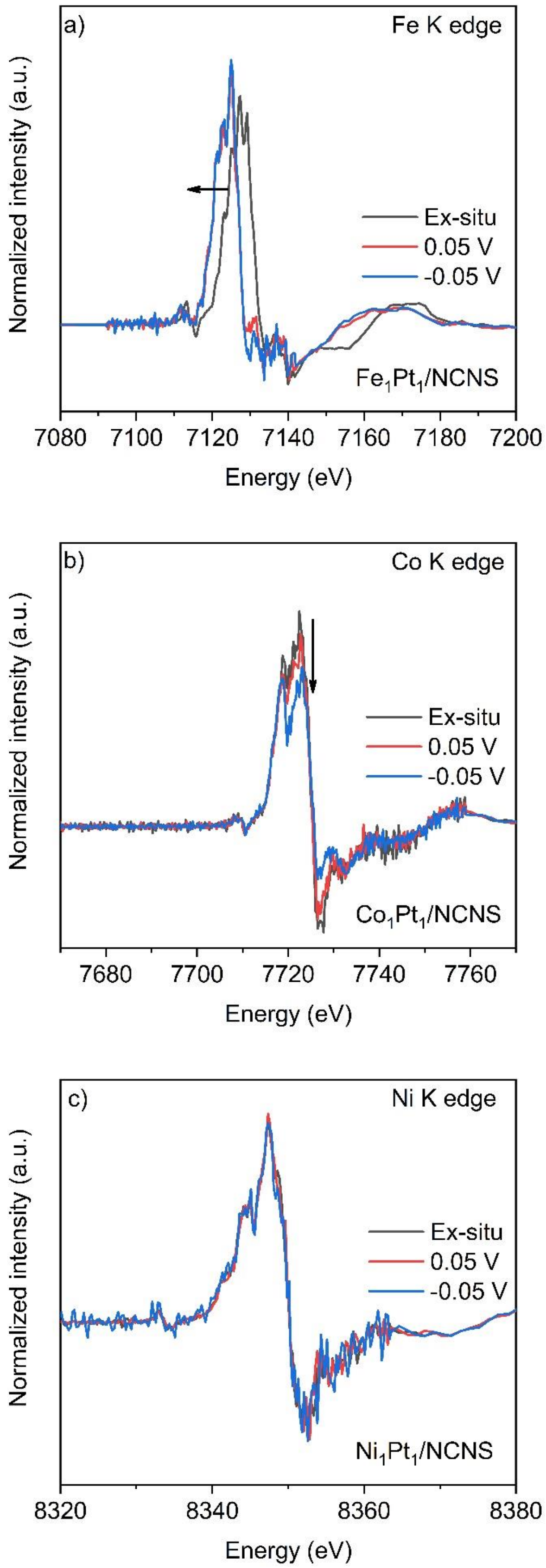

Supplementary Fig. 29 Derivative *operando* XANES spectra at the Fe (a) , Co (b) and Ni (c) *K* edges for Fe_1Pt_1/NCNS, Co_1Pt_1/NCNS, and Ni_1Pt_1/NCNS, respectively.

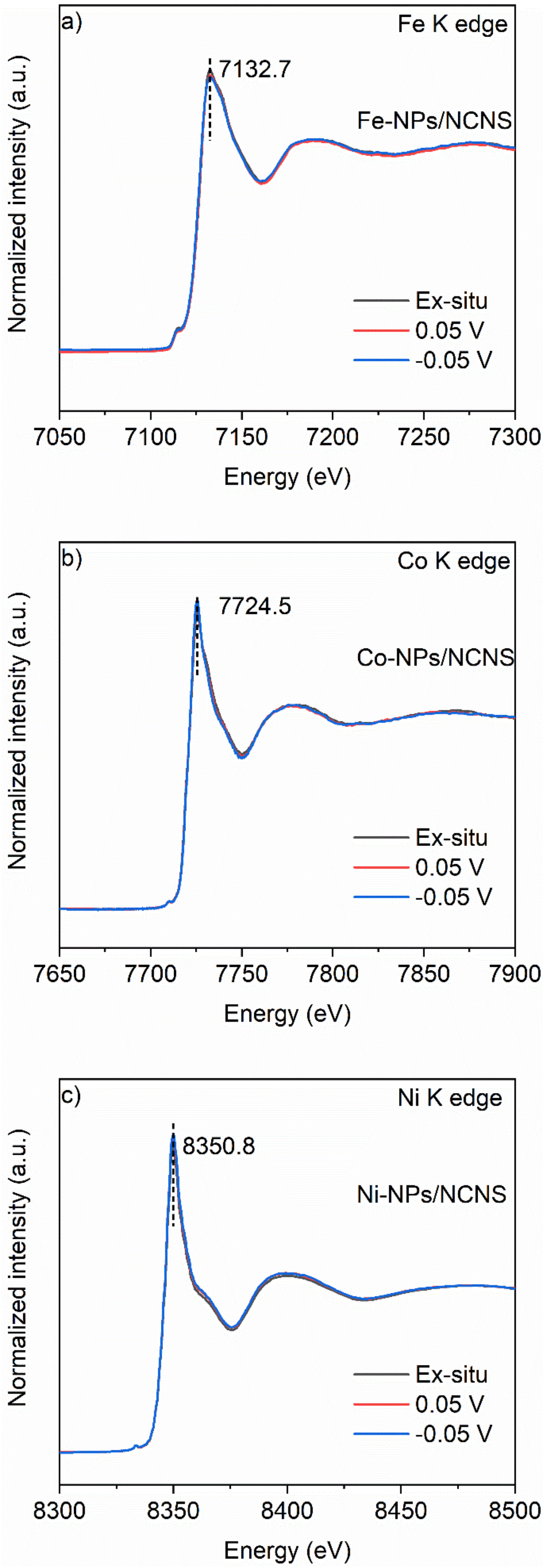

Supplementary Fig. 30 Operando XANES on Fe (a), Co (b), and Ni (c) -NPs/NCNS under HER condition at Fe, Co, and Ni *K* edges, respectively.

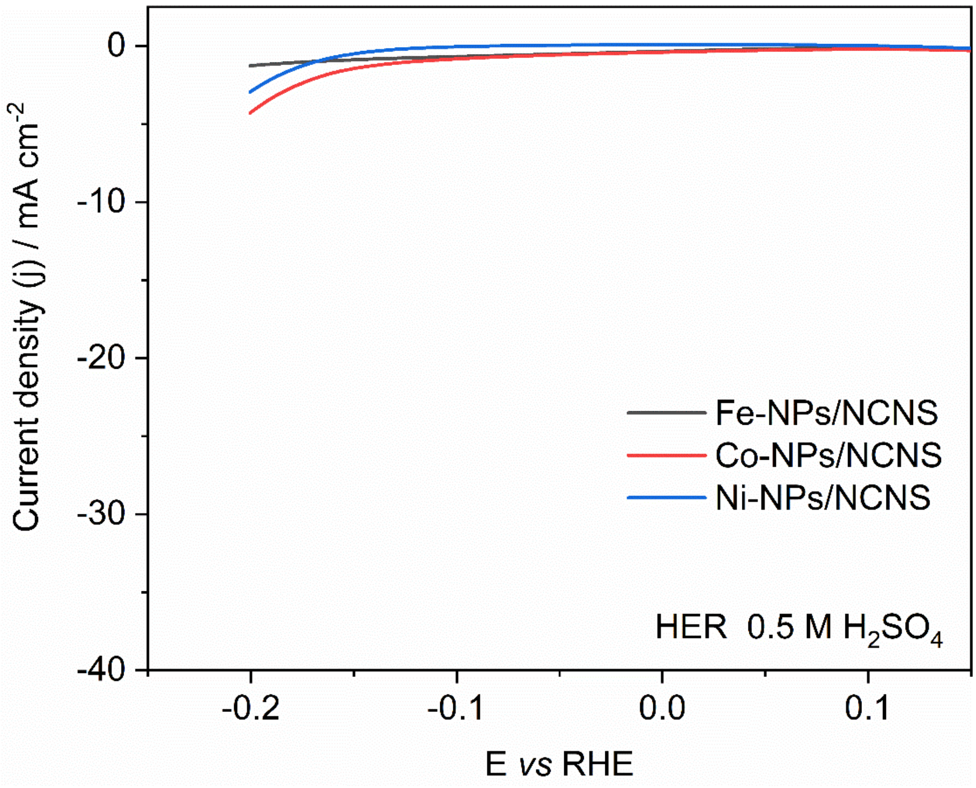

Supplementary Fig. 31 Electrocatalytic performance of Fe, Co, and Ni-NPs/NCNS in HER.

Supplementary Table 1. Structural parameters of the Co_1Pt_1/NCNS, Pt foil, and PtO_2 references extracted from quantitative EXAFS curve-fittings.

**Sample**	**Path**	**CNs**	***R*** **(Å)**	**σ^2 (10^{-3}Å^2)**	**ΔE_0 (eV)**
Pt foil	Pt-Pt	12.0	2.76	6.0	7.7
PtO_2	Pt-O	6.0	2.02	3.0	1.0
Co_1Pt_1/NCNS	Pt-C	2.1	1.98	1.3	4.8
	Pt-N	2.1	2.05	1.7	
	Pt-C	3.8	2.89	8.0	
	Pt-C	4.0	3.21	9.5	

CNs, coordination numbers; R, bonding distance; σ^2, Debye-Waller factor; ΔE_0, inner potential shift. Errors in the fitting parameters are CN ± 20%, R ± 0.02, σ^2 ± 20%, and ΔE_0 ± 3.0.

Supplementary Table 2. The comparison of EXAFS Fitting results and the optimized model of M_1Pt_1/NCNS (M = Co, Fe, and Ni).

Sample	EXAFS fitting		DFT	
	**Path**	***R*(Å)**	**Path**	***R*(Å)**
Co_1Pt_1/NCNS	Co-O	1.98	Co-O_1	1.93
			Co-O_2	1.94
	Co-N	2.08	Co-N	2.11
Fe_1Pt_1/NCNS	Fe-O	1.95	Fe-O_1	1.91
			Fe-O_2	1.92
	Fe-N	2.02	Fe-N	2.13
Ni_1Pt_1/NCNS	Ni-O	1.99	Ni-O_1	1.96
			Ni-O_2	2.00
	Ni-N	2.11	Ni-N	2.15

Supplementary Table 3. Calculated Bader charges of Co and two Cp rings of $Co(Cp)_2$ in the gas phase and absorption on Pt_1/NCNS

	**In gas**	**@ Pt_1/NCNS**
Co	+0.67	+0.68
Cp_1	-0.34	+0.10
Cp_2	-0.34	+0.01

Supplementary Table 4. Calculated *d*-band centre of metal atoms in M_1-pyrrolic N_4 catalysts. (unit: eV)

	**Spin up**	**Spin down**	**Average**
**Fe_1-pyrrolic N_4**	-1.97	0.07	-0.96
**Co_1-pyrrolic N_4**	-0.52	-1.56	-1.04
**Ni_1-pyrrolic N_4**	-1.38	-1.38	-1.38

Supplementary References

1 Lytle, F. W. Determination of d-band occupancy in pure metals and supported catalysts by measurement of the L_{III} X-ray absorption threshold. *J. Catal.* **43**, 376-379 (1976).

2 Fang, S. *et al.* Uncovering near-free platinum single-atom dynamics during electrochemical hydrogen evolution reaction. *Nat. Commun.* **11**, 1029 (2020).

3 Li, J. *et al.* Unveiling the Nature of Pt Single-Atom Catalyst during Electrocatalytic Hydrogen Evolution and Oxygen Reduction Reactions. *Small* **17**, 2007245 (2021).

4 Zhang, L. Z. *et al.* Charge Polarization from Atomic Metals on Adjacent Graphitic Layers for Enhancing the Hydrogen Evolution Reaction. *Angew. Chem. Int. Ed.* **58**, 9404-9408 (2019).

5 Zhang, L. Z. *et al.* Coordination of Atomic Co-Pt Coupling Species at Carbon Defects as Active Sites for Oxygen Reduction Reaction. *J. Am. Chem. Soc.* **140**, 10757-10763 (2018).

6 Zhuang, L. *et al.* Defect-Induced Pt–Co–Se Coordinated Sites with Highly Asymmetrical Electronic Distribution for Boosting Oxygen-Involving Electrocatalysis. *Adv. Mater.* **31**, 1805581 (2019).

7 Fei, H. *et al.* General synthesis and definitive structural identification of MN_4C_4 single-atom catalysts with tunable electrocatalytic activities. *Nat. Catal.* **1**, 63-72 (2018).

8 Montemore, M. M., van Spronsen, M. A., Madix, R. J. & Friend, C. M. O_2 activation by metal surfaces: implications for bonding and reactivity on heterogeneous catalysts. *Chem. Rev.* **118**, 2816-2862 (2017).

9 Liao, P., Keith, J. A. & Carter, E. A. Water oxidation on pure and doped hematite (0001) surfaces: Prediction of Co and Ni as effective dopants for electrocatalysis. *J. Am. Chem. Soc.* **134**, 13296-13309 (2012).
